# Relation of Gut Microbes and L-Thyroxine Through Altered Thyroxine Metabolism in Subclinical Hypothyroidism Subjects

**DOI:** 10.3389/fcimb.2020.00495

**Published:** 2020-09-18

**Authors:** Zhenyu Yao, Meng Zhao, Ying Gong, Wenbin Chen, Qian Wang, Yilin Fu, Tian Guo, Jiajun Zhao, Ling Gao, Tao Bo

**Affiliations:** ^1^Department of Endocrinology, Shandong Provincial Hospital, Cheeloo College of Medicine, Shandong University, Jinan, China; ^2^Shandong Key Laboratory of Endocrinology and Lipid Metabolism, Shandong Provincial Hospital, Jinan, China; ^3^Shandong Provincial Hospital Affiliated to Shandong First Medical University, Jinan, China; ^4^Central Laboratory, Shandong Provincial Hospital Affiliated to Shandong First Medical University, Jinan, China; ^5^Department of Ultrasound, Shandong Provincial Hospital Affiliated to Shandong First Medical University, Jinan, China

**Keywords:** subclinical hypothyroidism, Clinical Trails, L-Thyroxine treatment, gut microbiome, thyroid hormone metabolism

## Abstract

Thyroxine metabolism is an important topic of pathogenesis research and treatment schedule of subclinical hypothyroidism (SCH). L-Thyroxine replacement therapy (LRT) is usually recommended for severe SCH patients only. Our previous studies reported that disordered serum lipid of mild SCH people could also benefit from LRT. However, the benefits were different among individuals, as shown by the variations in drug dosage that required to maintain thyroid-stimulating hormone (TSH) stability. Alternative pathways, such as sulfation and glucuronidation of iodothyronine, may play a role in thyroid hormones metabolism in peripheral tissues aside from thyroid. Conjugated thyroxine can be hydrolyzed and reused in tissues including gastrointestinal tract, in which gut microbiota are one of the most attractive physiological components. On this site, the roles of gut microbiota in thyroidal metabolism should be valued. In this study, a cross-sectional study was performed by analyzing 16S rDNA of gut microbiota in mild SCH patients treated with L-thyroxine or not. Subjects were divided by serum lipid level, L-thyroxine treatment, or L-thyroxine dosage, respectively. Relationship between gut microbiome and serum profile, L-thyroxine treatment, and dose were discussed. Other metabolic disorders such as type 2 diabetes and hypertension were also taken into consideration. It turned out that microbiome varied among individuals divided by dose and the increment of L-thyroxine but not by serum lipid profile. Relative abundance of certain species that were associated with thyroxine metabolism were found varied among different L-thyroxine doses although in relatively low abundance. Moreover, serum cholesterol may perform relevance effects with L-thyroxine in shaping microbiome. Our findings suggested that the differences in L-thyroxine dosage required to maintain TSH level stability, as well as the SCH development, which was displayed by the increased L-thyroxine doses in subsequent follow-up, had relationship with gut microbial composition. The reason may due to the differences in thyroxine metabolic capacity in gut. In addition, the metabolic similarity of iodothyronines and bile acid in gut also provides possibilities for the correlation between host's thyroxine and cholesterol levels.

This study was registered with ClinicalTrials.gov as number NCT01848171.

## Introduction

Hypothyroidism is a common disorder that leads to secondary metabolic diseases, such as dyslipidemia (Minarikova et al., 2014). Depending on its severity, hypothyroidism is classified into two groups, overt hypothyroidism (OH) or subclinical hypothyroidism (SCH). SCH is defined as an elevated level of serum thyroid-stimulating hormone (TSH), with a normal range of thyroxine (T4) (Garber et al., 2012). According to TSH level, SCH was artificially divided into mild SCH (TSH < 10.0 mIU/L) and severe SCH (TSH ≥ 10.0 mIU/L). At present, the percentage of mild SCH patients is at least 75% of SCH (Cooper and Biondi, 2012; Liu et al., 2014), whereas L-thyroxine replacement therapy is usually recommended for severe SCH patients only, according to the guidelines for hypothyroidism. Mild SCH is considered as risk factor for dyslipidemia development and cardiocerebrovascular disease (Rotondi et al., 2010; McQuade et al., 2011; Gao et al., 2013). Until now, no therapeutic recommendations have been clearly defined due to limited evidence from randomized, controlled trials (Ochs et al., 2008; Garber et al., 2012; Pearce et al., 2013; Collet et al., 2014).

The mechanism of thyroxine metabolism is considered as the important topic of pathogenesis research and treatment schedule of SCH. It is a general agreement that sequential monodeiodination is the major mechanism in regulating the bioavailability of thyroid hormones *in vivo*. However, alternative pathways, such as sulfation and glucuronidation in liver, may play a role in thyroid hormones metabolism in peripheral tissues aside from thyroid (Wu et al., 2005). Those conjugations can esterify the phenolic hydroxyl group with sulfuric acid or glucuronic acid, which increasing the water solubility of iodothyronines, leading to elevated biliary and renal clearance and reduced intestinal absorption (Visser, 1996). Sulfoconjugation could markedly accelerate thyroxine deiodination to their inactive metabolites, reverse triiodothyronine (rT3) and T2, with nearly 200 times more than non-sulfated thyroxine (Wu et al., 2005). Besides, sulfated T4 completely blocks the outer ring deiodination to sulfated T3 (Kester et al., 2002). It means that once sulfated, thyroxine activity was inhibited by either transferring to inactive form or being retained in T4 form, unless the sulfate group were hydrolyzed. Glucuronidation of thyroxine prompts itself to excrete into the intestinal lumen through biliary flow. This translocation enhanced the elimination of thyroxine to feces, lower the hormone level, and downregulated its biofunction (Wu et al., 2005; Virili and Centanni, 2015). The increase in biliary T4 glucuronide (T4G) secretion can downregulate circulating T4 levels, in turn to stimulate TSH levels, resulting in thyroidal hyperplasia. In rats, several glucuronidation-stimulating treatment or drugs may lower T4 and T3 levels and provoke TSH secretion, even goiter (Wu et al., 1995, 2005).

Gut microbiota is one of the most attractive physiological component in the intestine, which show strong correlation with several diseases, especially metabolic disorders such as type 2 diabetes, hypertension, and dyslipidemia (Yang et al., 2015; Pedersen et al., 2016; Kolodziejczyk et al., 2019). In human and rats, it has been proven that a large amount of conjugated iodothyronines can be hydrolyzed in fecal suspension (Hazenberg et al., 1988). Considering that a sort of obligate anaerobic bacteria of gut microbial settlers possess glucuronidase activities, gut microbiome analysis might be focused in the research of thyroxine metabolism. Hydrolyzing of conjugated T4 in the gastrointestinal tract has provided convenience for the hormone to reenter the physiological circulation *via* hepatoenteral circulation and in turn join the iodothyronine pool (Hays, 1988). These microbial activities are somewhat similar to their roles in cholesterol metabolism. It was reported that gut flora were involved in cholesterol and bile acid metabolism especially in the deconjugation of conjugated bile acid (Ge et al., 2018). Bile acid sulfatase activity had been detected in intestinal isolates, which belong to *Pseudomonas* (Gerard, 2013; Bo et al., 2017). Gut microbiota may serve as a potential therapeutic target in regulating cholesterol disorders. On this site, the important roles of gut microbiota in thyroidal metabolism should be valued.

Moreover, the bioactivity of a drug is first dependent on its capacity of crossing the intestine barrier. Besides serving as the hydrolase executor, gut microbiota can effectively influence the permeability and integrity of the intestine barrier (Desai et al., 2016). Some researchers even considered gut microbiota as part of the intestine barrier. In germ-free mice, gut microbiota deficiencies can reduce the intestinal surface area, which reflects as shorter villi and decreased intestinal crypts (Natividad and Verdu, 2013). From this perspective, the diversity and structure of gut microbiota may act multifactorial roles in regulating the drug-controlled thyroidal metabolism.

Those multiple physiological functions of intestinal microorganism in thyroxine metabolism, including acting as hydrolase executor and intestinal barrier builder, had aroused great interests. The correlation of thyroid function and gut microbiota was built by several researchers (Hazenberg et al., 1988; McQuade et al., 2011; Zhou et al., 2014; Virili and Centanni, 2015; Feng et al., 2019). Other than the studies that agreed that thyroid disorders are the causal factor in the relationship with gut microbes, some researchers had indicated that bacteria might act as the motivating factor, as thyroid function may be impaired in patients with small intestinal bacterial overgrowth (SIBO) (Konrad et al., 2018). However, the causative role of thyroid and gut microbiota was not thoroughly ascertained yet.

Since 2013, an open-label, randomized, controlled trial was performed in Ningyang County, China (*LRT-2013*, Cinical Trails Registry No. NCT01848171), with the aim to illustrate the detailed effects of L-thyroxine replacement therapy in the improvement of thyroid function and lipid profiles of SCH patients. It turned out that mild SCH patients could benefit from L-thyroxine replacement therapy, shown as ameliorated thyroid autoimmunity, improved thyroid function, and improved lipid profiles (Zhao et al., 2016). The survey, which is a cohort study, required us to follow the TSH levels of the participants and adjust the dose of L-thyroxine on schedule. We found that, to maintain TSH within the normal range, the doses of the drug that we gave to the L-thyroxine treatment group were obviously varied among individuals (from 25 to 125 μg/day), according to their TSH level. These individual discrepancies might be caused by several factors, such as age, body mass index (BMI), glucose, and lipid profile. Considering that the intestine is the “second important reservoir” of iodothyronines, just after the thyroid gland (Wu et al., 2005), in which gut microbiota play significant potential roles, this cross-sectional study was performed by focusing gut microbiota to explore the possible influencing factors in causing thyroxine metabolism discrepancies. The possible factors such as dyslipidemia, type 2 diabetes (T2D), and hypertension were taken into consideration. Among them, the differences between individual's metabolism and absorptive capacity of L-thyroxine may contribute to the maximum effect.

## Materials and Methods

### Participants

The cross-sectional trial in this study, which was performed in June 2017, is one of the follow-ups of the open-label, randomized, controlled trial (*LRT-2013*) conducted in Ningyang County. Subjects enrolled in *LRT-2013* diagnosed as mild SCH (4.2 < TSH ≤ 10.0 mIU/L) were randomly divided into intervention group and control group, to receive either L-thyroxine (Merck KGaA, Darmstadt) replacement therapy (LRT group) or no treatment (NC group). All subjects were under follow-up. Serum FT3, FT4, TSH level, as well as other serum biochemical indicators were measured on schedule, and the doses of L-thyroxine applied in individuals of LRT group were adjusted by a clinician according to the thyroid function (serum TSH, FT3, FT4 level). In this study, we performed a cross-sectional research of gut microbiome analysis in the survey schedule of June 2017, with exclusion criteria mentioned in our previous study (Zhao et al., 2016).

### Sample Collection and Grouping Criteria

Enrolled SCH subjects from both groups (NC and LRT group) were required to have fasted overnight. In the following morning, they were told to defecate; fecal samples were collected by using a stool collecting tube and stored in −80°C. Apart from this, the general information and blood sample of all subjects were collected for further medical examination, including age, body weight, TSH, T4, serum triglyceride (TG), serum cholesterol (TC), and high- (HDL) and low-density lipoproteins (LDL), and a self-report questionnaire was completed by each individual. The doses of L-thyroxine were adjusted according to the individual's TSH level. Based on the information from the self-report questionnaire, we made a further exclusion by virtue of additional criteria as follows: (1) any subject who did not operate as required during sample collection; (2) taking any medicine in the previous 3 months that affects gut microbiota, including antibiotics and probiotics; and (3) any gastrointestinal diseases in the previous 6 months, including irritable bowel syndrome (IBS), small intestinal bacteria overgrowth (SIBO), etc. (Nicolucci et al., 2017; Grimaldi et al., 2018). Based on the lipid profile, all subjects were subsequently divided into SCH and high triglyceride (STG) group (TG ≥ 1.7 mmol/L, TC < 5.2 mmol/L), SCH and high cholesterol (STC) group (TC ≥ 5.2 mmol/L, TG < 1.7 mmol/L), SCH and high triglyceride and high cholesterol (SM) group (TG ≥ 1.7 mmol/L, TC ≥ 5.2 mmol/L), and the group with normal serum lipid profile (S) (TG < 1.7 mmol/L, TC < 5.2 mmol/L). The grouping criteria was according to the guideline for Chinese adult dyslipidemia prevention and treatment (2016 Edition). In addition, based on the dose of L-thyroxine, subjects in the LRT group were manually divided into low dose (L) group, middle dose (M) group, and high dose (H) group. Briefly, according to advices from clinicians, subjects who had <50 μg/day (50 μg is not included) in the past 3 months were assigned into the L group, those who had 50–75 μg/day (75 μg is not included) were assigned into the M group, and those who had more than 75 μg (up to a maximum of 125 μg) were assigned into the H group. Moreover, during the follow-up study of *LRT-2013*, the doses of L-thyroxine applied in individuals in the LRT group were adjusted on schedule according to the thyroid function. In our study, besides our survey in June 2017, we also collected the L-thyroxine dosage data of one follow-up in March 2018 (9 months after the fecal sampling time). Based on the follow-up data, subjects in the LRT group were subdivided into developing (L-D) group and no develop (L-ND) group depending on whether the dose was increased or not on March 2018.

Since other metabolic disorders, such as T2D and hypertension, had certain population and might influence clinical and microbial profiles, cases after removing those who take diabetes and/or hypertension medication were subsequently reanalyzed to get clearer information, although it lowered the sample size. Diabetes was diagnosed based on the World Health Organization (WHO) 1999 criteria, as FPG concentration ≥7.0 mmol/L and/or self-reported history of T2D (Alberti and Zimmet, 1998). Diagnosis of hypertension was defined as self-reported treatment for hypertension with antihypertensive medications or a systolic BP ≥ 140 mmHg or diastolic BP ≥ 90 mmHg according to the 2010 Chinese guidelines (Liu and Writing Group of Chinese Guidelines for the Management of Hypertension, 2011).

### DNA Extraction and High-Throughput Sequencing

Total DNA from fecal sample was extracted by cetyltrimethyl ammonium bromide (CTAB)/ sodium dodecyl sulfate (SDS) method as previously reported (Bo et al., 2017) and qualified by a UV spectrophotometer (Thermo Nanodrop 2000) and agarose gel electrophoresis. The 16S ribosomal DNA (rDNA) V3–V4 region was selected and amplified by degenerate primers (341F: 5′-CCTAYGGGRBGCASCAG-3′; 806R: 5′-GGACTACNNGGGTATCTAAT-3′), with index sequences and adaptors. PCR was performed by KAPA HiFi Hotstart ReadyMix PCR Kit. PCR products were detected by electrophoresis using 2% agarose gels, and samples in the range of 400–450 bp were excised and extracted using a gel extraction kit (Axyprep, Axygen) for further analysis.

16S rDNA libraries were generated by a NEB Next® UltraTM DNA Library Prep Kit for Illumina (New England Biolabs, USA). Libraries were qualified on the Qubit^@^3.0 Fluorometer (Thermo Scientific) and Agilent Bioanalyzer 2100 system. The eligible libraries were sequenced on an Illumina Hiseq PE250 platform.

### Data Analysis

After quality control (QC), original data were filtered and clustered by Usearch. Reads were assigned to each sample using the unique index. Based on homologous alignment analysis, sequences with ≥97% similarity were assigned the same taxonomic unit, which was defined as optimal taxonomic unit (OTU). One representative sequence was chosen in each OTU for taxonomic information annotation. Alpha diversity analysis was performed to analyze the diversity in a single sample, which includes observed species index, Chao1 index, and Shannon and Simpson index. Observed species indicate the actual OTU numbers observed according to the sequencing results; Chao1 index is used to estimate the total OTU number contained in each sample. The Shannon and Simpson index was used to estimate the diversity of microbial community; higher Shannon and Simpson index indicate higher diversity. The corresponding dilution curves were calculated and drawn by QIIME (Kemp and Aller, 2004; Caporaso et al., 2010), based on the relative proportions of each OTU. GraphPad software was responsible for drawing the curves and box plot. Leveling parameters were set according to the observed species curve. All OTUs were subsequently analyzed for abundance and diversity. For beta diversity, principal component analysis (PCA) and principal coordinates analysis (PCoA) were performed to dimensionally reduce and simplify the OTU variations among samples by using R project, which enables the multidimensional differences to be reflected as the distance between dots on the two-dimensional coordinate graph. The coordinate axes are set by the first two eigenvalues with largest contributions to the differences. Different colors represent samples belonging to different groups. Samples between different groups may exhibit a distribution of dispersion or aggregation so that it is possible to judge whether the sample compositions have similarities. The further the distance between the two dots, the greater the difference in microbial community between the two samples; the closer the distance between the two points, the more similar the microbial community composition of the two samples. Correlations between microbial community members and environmental factors were calculated using canonical correspondence analysis (CCA). PCA was performed by STAMP v2.1.3; PCoA was performed by R project (R 3.6.3). CCA was performed by Canoco v5.0.

### Statistical Analysis

All statistical analyses were performed using SPSS version 22.0 for Windows (Chicago, IL, USA). For the basal differences of serum lipid profile among groups (normally distributed numerical variables), Student's *t*-test was used for two-group comparisons, and one-way ANOVA test was used for three- or four-group comparisons. For comparisons between different taxonomies (levels of genus, family, etc.) among different groups within each subgroup, Mann–Whitney *U*-test was used for two-group comparisons, and Kruskal–Wallis *H*-test was used for three-group comparisons of non-normally distributed numerical variables.

### Ethics Statement

Our research was approved by the Ethics Committee of Shandong Provincial Hospital, and the methods were performed according to the approved guidelines. This study was registered with ClinicalTrials.gov as number NCT01848171.

## Results

### Basal Clinical Characteristics of the Study Population

**Figure 1:**
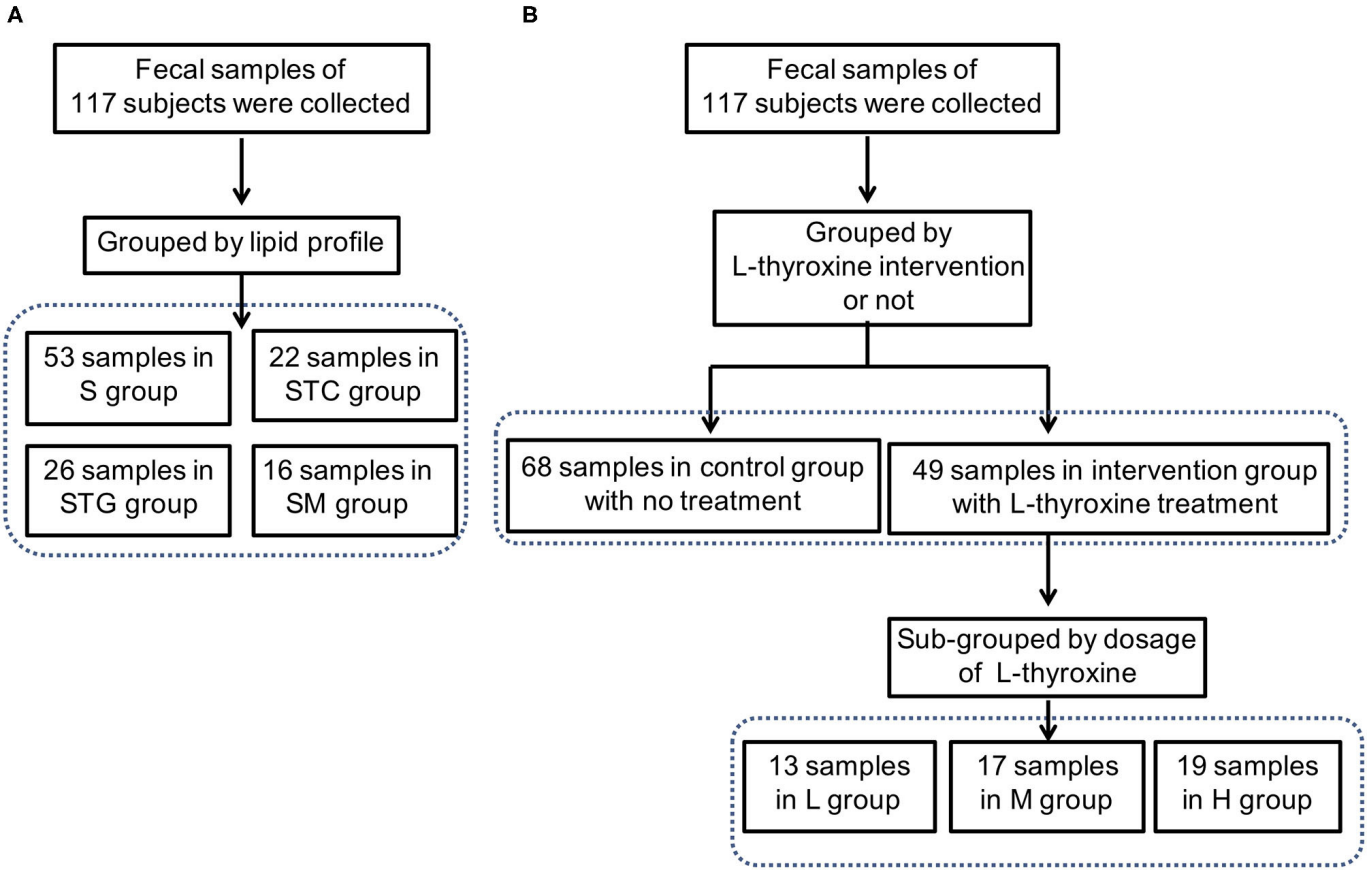
Trial profile. **(A)** All subjects were divided into the STG [subclinical hypothyroidism (SCH) with high serum triglyceride level] group, STC (SCH with high serum cholesterol level) group, SM (SCH with both high serum triglyceride and cholesterol level) group, and S (SCH with normal serum lipid profile) group according to the guidelines of Chinese adult dyslipidemia prevention and treatment (2016 edition). **(B)** All subjects were divided into L-thyroxine replacement therapy (LRT) group and NC group. In addition, subjects in LRT group were manually subdivided into low dose (L) group, middle dose (M) group, and high dose (H) group based on the doses of L-thyroxine.

Three hundred twelve fecal samples were collected from mild SCH subjects. The doses of L-thyroxine data were statistically sorted, and 195 subjects were subsequently excluded due to missing data at a certain time point (especially the subjects under L-thyroxine treatment, for large amount of people who did not take the medicine on time or were lost in touch at the time when the drugs were distributed). Final 117 samples were involved, in which 49 of them are from the L-thyroxine replacement therapy (LRT) group (10 cases with confirmed T2D); the remaining 68 were from the NC group (20 cases with confirmed T2D). Based on the guideline for Chinese adult dyslipidemia prevention and treatment (2016 Edition), all subjects were subsequently divided according to lipid profile, in which 26 subjects were classified into the STG group, 22 into the STC group, 16 into the SM group, and the rest 53 into the S group (Figure 1A). Moreover, based on L-thyroxine doses, subjects within the LRT group were manually subdivided into L group (13 subjects), M group (17 subjects), and H group (19 subjects) (Figure 1B). In addition, basal physiological data such as age, BMI, as long as serum biochemical characters, including TSH, T4, serum triglyceride (TG), serum total cholesterol (TC), HDL, LDL, and blood pressure are listed in Table 1. The FPG and blood pressure level are slightly higher but still within normal ranges. The information of the population after excluding T2D and/or hypertension medication cases are summarized and listed in Supplementary Table 1.

**Table 1:** Basal physiological data analysis according to different grouping criteria.

**Group**	**Age**	**Gender**	**BMI**	**DM, (%)**	**SBP**	**DBP**	**FPG**	**ALT**	**AST**	**TG**	**CHOL**
	**(year)**		**(kg/m^**2**^)**	**(%)**	**(mmHg)**	**(mmHg)**	**(mmol/L)**	**(U/L)**	**(U/L)**	**(mmol/L)**	**(mmol/L)**
LRT	64.1 ± 7.8	10/39	25.2 ± 3.3	6 (34.7%)	130.9 ± 20.0	77.9 ± 10.7	5.90 (1.07)	20.0 (10.0)	26.0 (9.0)	1.50 (1.40)	5.54 ± 1.05
NC	63.9 ± 8.6	18/50	26.0 ± 3.5	15 (22.1%)	135.7 ± 19.1	80.8 ± 14.9	5.91 (1.28)	18.0 (10.0)	26.0 (7.8)	1.42 (0.98)	5.20 ± 1.04
S	63.9 ± 9.2	18/35	26.4 ± 3.7	7 (13.2%)	135.3 ± 20.0	80.4 ± 16.1	5.74 (0.80)	18.0 (10.5)	25.0 (8.0)	1.18 (0.58)	4.91 ± 0.77
STC	65.7 ± 7.4	2/20	24.8 ± 2.4	1 (4.5%)	131.7 ± 20.5	76.3 ± 10.4	5.85 (0.94)	16.0 (9.0)	26.5 (7.5)	1.26 (0.44)	6.32 ± 0.66^*^
STG	64.2 ± 7.0	5/21	25.7 ± 3.6	8 (30.8%)	135.4 ± 18.7	81.9 ± 10.0	6.29 (2.87)^*^	19.0 (9.5)	24.5 (9.5)	2.33 (1.46)^*^	4.66 ± 0.81
SM	61.5 ± 8.4	3/13	24.0 ± 2.5	5 (31.3%)	128.5 ± 18.4	77.4 ± 11.9	6.55 (2.37)^*^	23.0 (13.5)	30.0 (11.0)	2.67 (2.22)^*^	6.50 ± 0.71^*^
H	64.5 ± 7.7	5/14	25.4 ± 3.0	5 (26.3%)	137.1 ± 23.0	77.9 ± 6.7	6.10 (2.86)	20.0 (11.0)	27.0 (12.0)	1.51 (1.81)	5.75 ± 1.15
M	64.4 ± 8.9	3/14	25.3 ± 3.5	0	124.1 ± 17.3	76.4 ± 12.8	6.00 (0.67)	18.0 (13.5)	24.0 (9.0)	1.50 (1.40)	5.43 ± 0.90
L	64.2 ± 7.3	2/11	24.6 ± 3.7	1 (7.7%)	129.9 ± 15.1	78.3 ± 11.9	5.80 (0.86)	20.0 (8.0)	22.0 (9.0)	1.47 (1.15)	5.51 ± 1.13
L-D	64.2 ± 8.1	6/31	24.8 ± 3.0	3 (8.1%)	129.8 ± 17.6	76.7 ± 10.2	5.90 (0.92)	19.0 (9.5)	24.0 (9.5)	1.50 (1.22)	5.51 ± 1.05
LND	65.2 ± 7.4	4/8	26.5 ± 4.1	3 (25.0%)	133.7 ± 26.0	80.0 ± 10.9	6.44 (2.32)	20.0 (10.5)	27.0 (8.5)	1.55 (1.73)	5.75 ± 1.08

*Gender, data was shown as male/female*.

*BMI, body mass index; DM, type 2 diabetes millitus; FPG, fasting plasma glucose; TG, serum triglyceride; CHOL, serum cholesterol; AST, glutamic-pyruvic transaminase; ALT, glutamic oxaloacetic transaminase*.

* *p < 0.01 within their respective groups*.

### Overall Information of Total Sequencing Data and the Quality Control

Total DNA were extracted from fecal samples, 16S rDNA was amplified, and high-throughput sequencing was performed by Illumina Hiseq platform to assess the microbial profile. The overall information of samples and sequencing data were summarized. On average, the value of Q20 and Q30 were 94.8 and 91.3%, respectively. Clean reads (Kemp and Aller, 2004; Desai et al., 2016) were collected in each sample, with an average length of 415 bp. After mapping, 30,753 clean reads were obtained, with the ratio of 86.1%. Due to the total amount of clean reads varied among samples, the species abundance, which is displayed by the amount of certain specific reads, may be affected by the deviation of each sample's total amount. Higher quantity of certain species in a certain sample may not reflect the higher absolute abundance of the species, but due to the higher total clean reads, a sample was obtained. Under the premise that the sequence depth is sufficient, to minimize these deviations among samples, the clean reads of each sample were randomly downsized to 21,633, with average OTUs downsized from 197 to 184 per sample.

### Alpha Diversity Shows No Obvious Difference in Species Diversity Among Groups

Observed species analysis, Chao1 analysis, as well as Shannon and Simpson analysis were performed to test the sufficiency of the sequence collection and the microbial diversity of the samples (Supplementary Figure 1). From Supplementary Figures 1A,B, the observed species and Chao1 index (Y-axis) showed a gradual slow increase tendency along with the increase in sequences number (X-axis) and displayed as a plateaued shape at the end of the curve, which indicated that enough sequences were obtained to cover the majority of species. Shannon and Simpson indexes showed no statistical differences among those groups with serum lipid disorders, which indicated that the microbial diversity did not change obviously according to the serum lipid profile (Supplementary Figures 1C,D), either in the comparison of NC or LRT group (Supplementary Figure 2). These results had demonstrated that the sequencing depth was enough for our further analysis. However, the microbial diversity was not influenced by combined lipid disorders (triglyceride and cholesterol) or T4 intervention.

### Gut Microbial Profile Showed Correlations With L-Thyroxine Dose in Beta-Diversity Analysis

**Figure 2:**
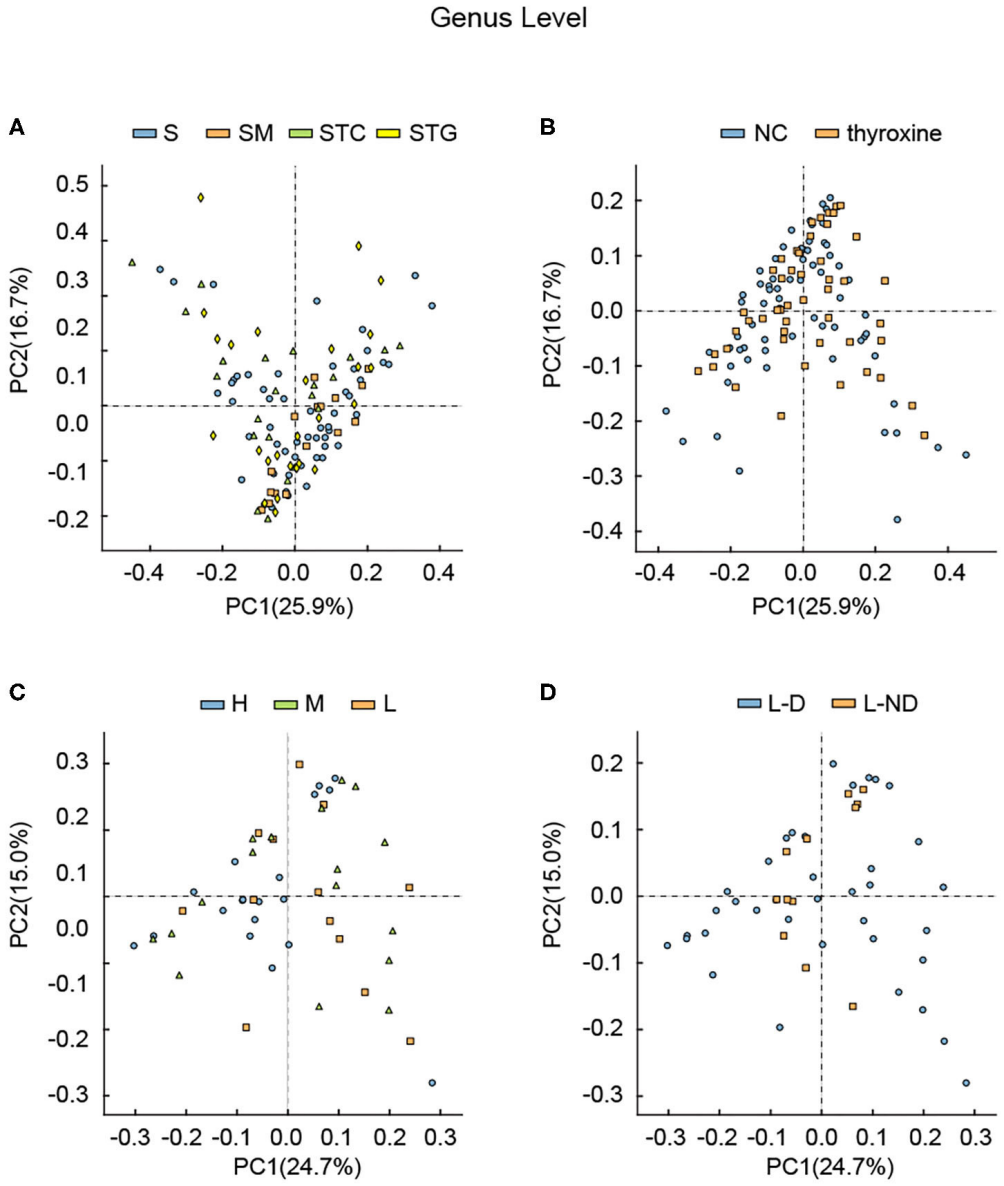
Principle component analysis among different groups divided by **(A)** lipid profile, **(B)** with or without L-thyroxine treatment, **(C)** L-thyroxine dosage within the LRT group, and **(D)** the development of L-thyroxine dosage within the LRT group in genus levels. X- and Y-axes represent the first principal component (PCA1) and the second principal component (PCA2), respectively. The percentage in the brackets represents the relative contribution of the component to the total difference. Each sample corresponded to one dot in the graph. Different group is represented by different color.

In order to illustrate the possible relationship between lipid profile, L-thyroxine treatment, and gut microbiota in SCH patients, we further analyzed the beta diversity discrepancies among different groups according to different types of grouping criteria. PCA and PCoA were performed in each taxonomic level. The microbial discrepancies were calculated and dimensionally reduced to two components. The differences between samples were intuitively displayed as the distance between points. In PCA, there was no obvious difference among groups divided by lipid profile in genus level (Figure 2A) and other taxon (Supplementary Figure 3). Interestingly, the samples in the SM group were slightly different compared with other samples, as they showed a tendency to gathering together. These results indicated that in SCH patients, the disorders of triglyceride and cholesterol worked cooperatively, leading to the relatively similar composition and stable structure of the microbial community.

**Figure 3:**
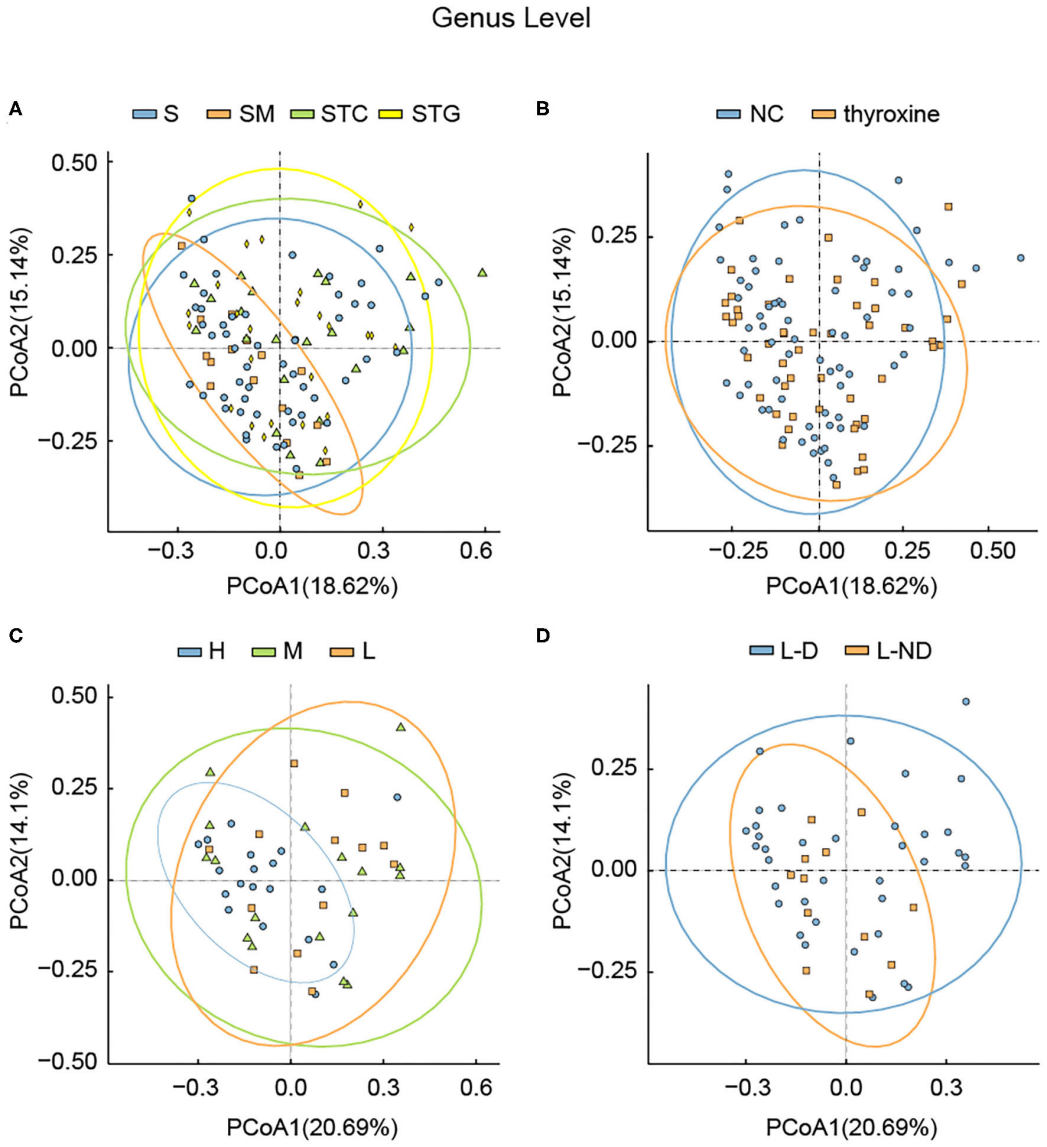
Principle coordination analysis among different groups divided by **(A)** lipid profile, **(B)** with or without L-thyroxine treatment, **(C)** L-thyroxine dosage within the LRT group, and **(D)** the development of L-thyroxine dosage within the LRT group in genus levels. X- and Y-axes represent the first principal component (PCoA1) and the second principal component (PCoA2), respectively. The percentage in the brackets represents the relative contribution of the component to the total difference. Each sample corresponded to one dot in the graph. The circle summarized the area of gathering of the dots. Different groups, together with the circle, are represented by different colors.

Considering that several studies had focused on the microbial difference analysis of L-thyroxine treatment, we further performed analysis between NC and LRT groups, divided by L-thyroxine treatment or not, and H, M, and L groups, divided by the doses of L-thyroxine. Given the fact that the species diversity showed no obvious change among groups of L-thyroxine treated or not, or with different L-thyroxine doses, which were shown by Shannon and Simpson index (Supplementary Figures 2, 4), samples between NC and LRT groups did not show discrepancies with statistical significance by PCA (Figure 2B, Supplementary Figure 5), whereas the samples in high doses of L-thyroxine group (H group) exhibited a clustered tendency, especially in PC1 axis of genus and family levels (Figure 2C, Supplementary Figure 6). These results showed that L-thyroxine treatment did not affect much the shaping gut microbial profile, as subjects in the LRT group did not show much differences compared with the control group. The potential possible influence of T2D and/or hypertension to gut microbial was taken into consideration. Aside from total population, same analysis was performed after excluding T2D and/or hypertension medication cases; the results showed that there were no obvious changes in microbial profile after excluding (Supplementary Figure 7). Interestingly, in PCA, within LRT subjects, those who need large amount of the drug shared a relatively similar gut microbial profile, which can be partially speculated that in the correlation between gut microbiota and thyroxine metabolism, bacteria may act as the causal factor. However, the differences among groups in PCoA were not as obvious as that in PCA, both in total population (Figure 3) and after removing T2D and/or hypertension medication cases (Supplementary Figure 8). Together, these interesting phenomena prompted us to seek for some more evidence to clarify the causal relationship between gut microbes and thyroxine metabolism.

### Gut Microbes Might Involve in SCH Development

**Figure 4:**
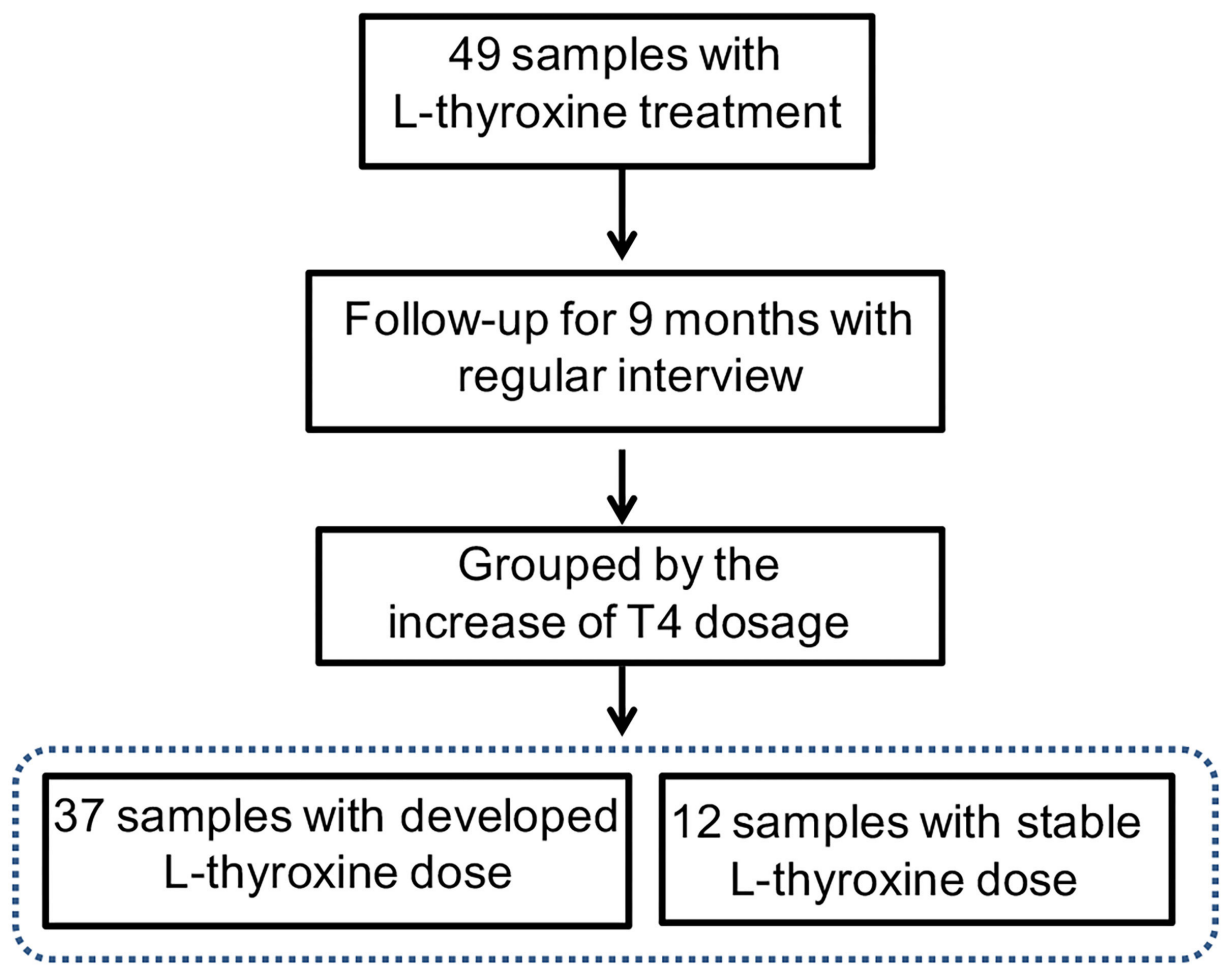
Trial profile. Subjects in the L-thyroxine replacement therapy (LRT) group were manually subdivided into L-D (L-thyroxine dose increased) and L-ND groups (L-thyroxine did not increase) based on whether the dose had increased during the last 9 months or not.

In our study, subjects underwent physical examination on a regular basis. The dose of L-thyroxine was adjusted on schedule, according to the TSH level. Considering the possible influences of gut microbiota in mediating thyroxine metabolism, SCH development of subjects in LRT group was focused to make clear whether the microbial profiles have correlations with the development of SCH. The adjusted doses of L-thyroxine were collected at the time point of 9 months later (March, 2018) than the sampling time (Jun, 2017). The development of SCH was defined depending on whether the dose had increased during the last 9 months or not, which was subsequently used for dividing the samples into L-D and L-ND groups (Figure 4). Analysis of the stool sample was performed to build the correlation with the SCH developing. As shown, samples with stable L-thyroxine dose in the further 9 months showed a relatively stable microbial “enterotypes,” which displayed a clustered tendency, in PCA analysis of total population (Figure 2D, Supplementary Figure 9) and the population after excluding T2D and/or hypertension medication cases (Supplementary Figure 7D), especially in the PC1 axis. However, the analysis in PCoA did not show obvious differences (Figure 3, Supplementary Figure 8) among groups divided by different criteria, either before or after excluding medication cases. Although these discrepancies are not particularly obvious, based on the fact that gut microbes have the abilities to metabolize thyroxine, it could be a hypothesis that gut microbes might affect the occurrence and development of SCH. The relative abundance of certain species that take part in the thyroxine metabolism should be further analyzed to provide detailed evidence for the hypothesis.

### Relative Abundance Analysis of Certain Bacteria Associated With Thyroxine Metabolism

**Figure 5:**
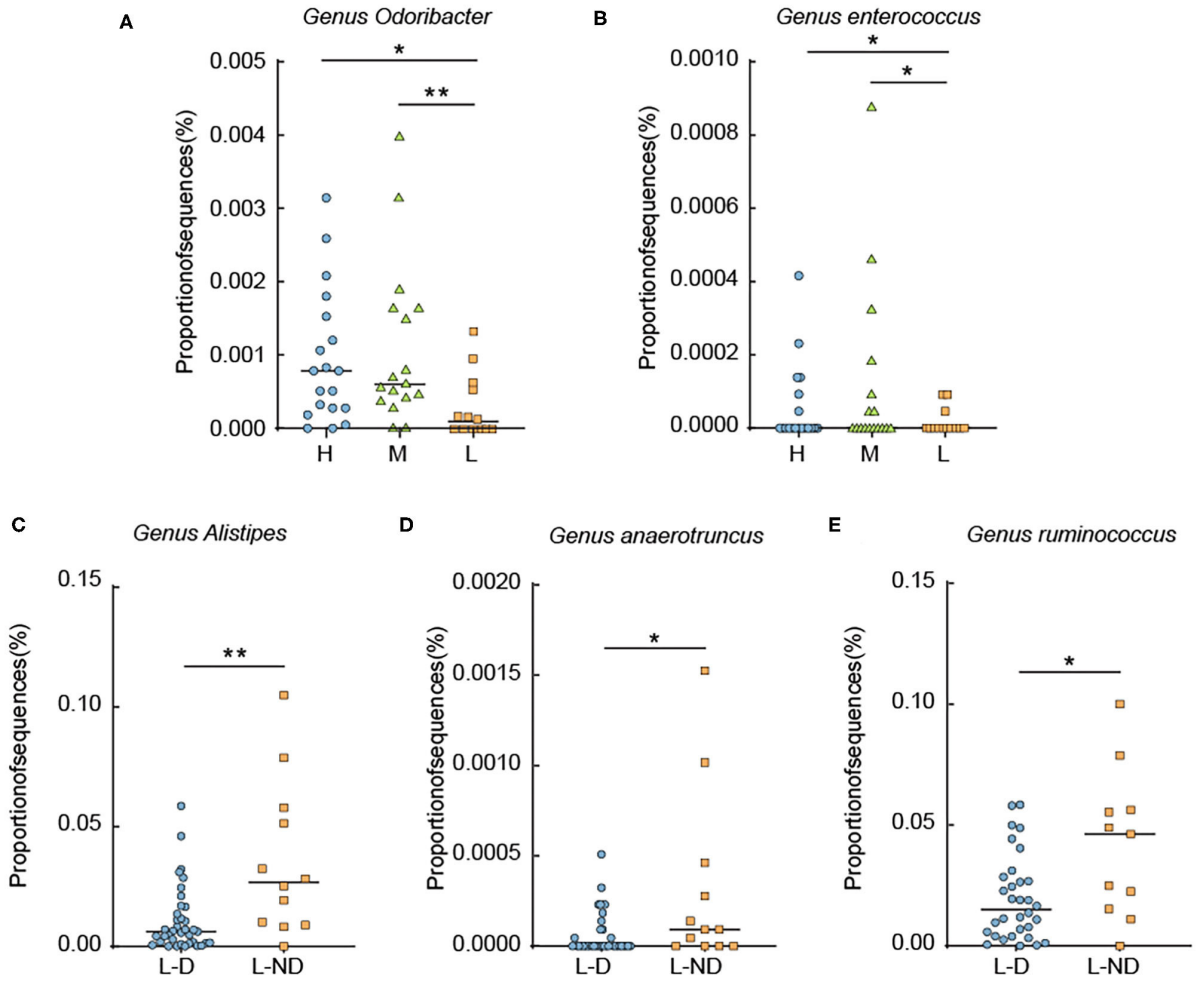
Relative abundance analysis of some metabolic representative species. **(A,B)** Relative abundance of the genera *Odoribacter* and *Enterococcus* among groups divided by L-thyroxine dosage. **(C–E)** Relative abundance of the genera *Alistipes, Anaerotruncus*, and *Ruminococcus* divided by L-thyroxine dosage development within the LRT group. Error bars are calculated as a standard error (SEM). The differences among groups were compared using nonparametric tests. *p < 0.05, **p < 0.01, defined statistically significance.

To identify the detail discrepancies associated with L-thyroxine dosage and SCH development, the relative abundances of certain bacterial taxa were analyzed. First, microorganisms that had reported associating with thyroxine metabolism were calculated. The relative abundance of the genera *Odoribacter* and *Enterococcus* was changed according to the dosage of L-thyroxine. The relative abundance of the genera *Alisipes, Ruminococcus*, and *Anaerotruncus* showed discrepancies with statistical significance between L-D and L-ND groups, both before (Figure 5) and after removing T2D and/or hypertension medication cases (Supplementary Figure 10). These species, although in low abundance, more or less with the hydrolytic activities or carbohydrate metabolic activities, may be involved in the metabolism of glucuronidated and sulfated iodothyronines or with the function of regulating the intestinal barrier.

Interestingly, among the species that show discrepancies between groups, some of them had been reported to be involved in bile acid metabolism in the gut. Genus *Odoribacter* had been reported with a negative correlation with lipid profiles especially cholesterol level. Genus *Alistipes* was involved in bile acid metabolism (Huang et al., 2019). The possible internal connection might be that the hydrolysis of glucuronidated and sulfated iodothyronines and the hydrolysis of conjungated bile acid share the similar metabolic process in the gut. The potential correlation of microbiota and environmental factors was further analyzed.

### Serum Cholesterol May Perform Relevance Effects With L-Thyroxine in Shaping Gut Microbial Profiles

**Figure 6:**
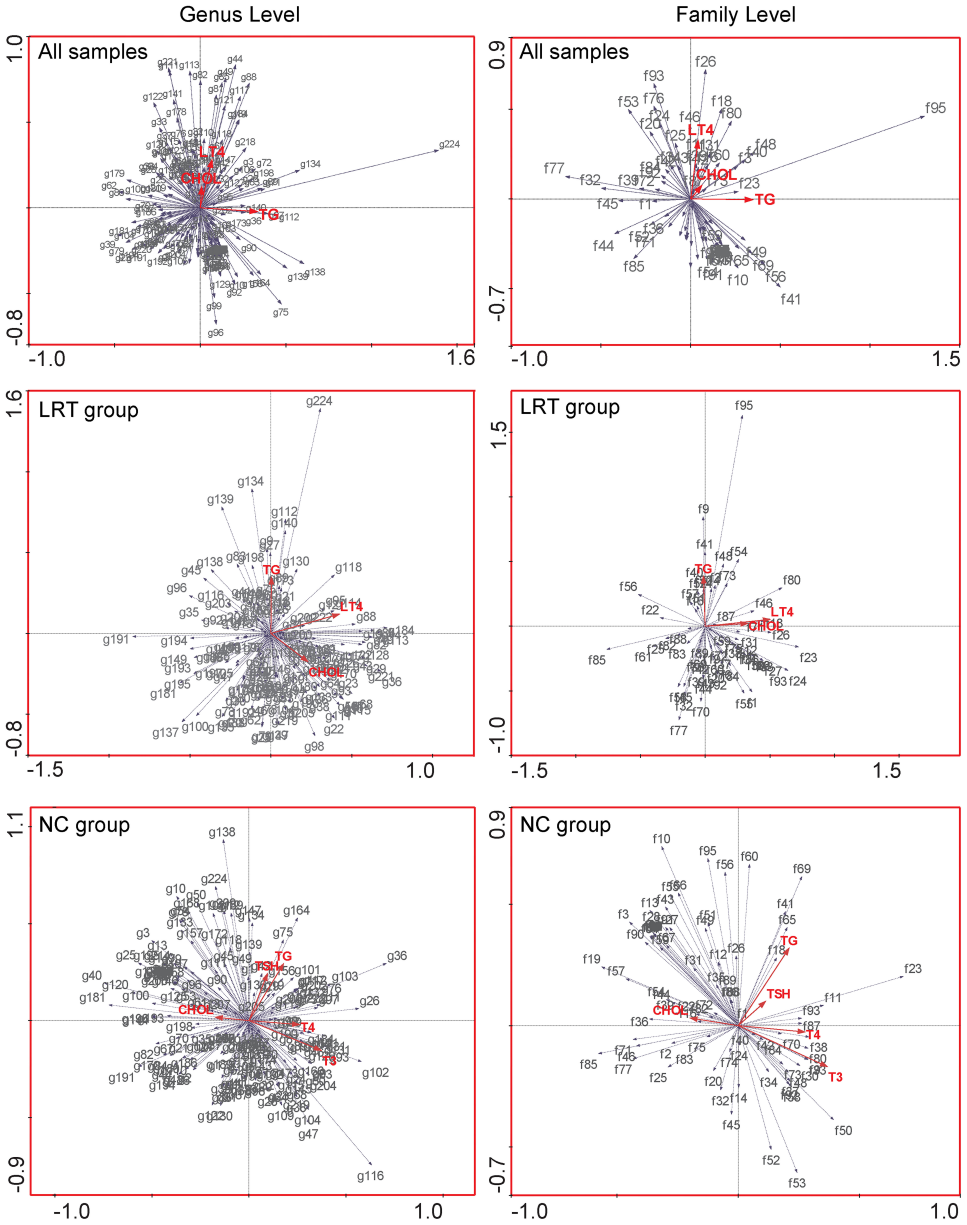
Canonical correspondence analysis (CCA) was used to evaluate the possible association of gut microbes with environmental factors, in all samples (upper row), LRT groups (middle row), and NC group (lower row), respectively. The red arrows in the figure represent different environmental factors, gray arrows were set pointing to species, and the length of the gray arrows quantitatively indicated the correlation significance between certain species and environmental factors. The angle between any two arrows is representative of the correlation between certain species or species and environmental factors. The acute angle indicates positive correlation, and the obtuse angle is a negative correlation.

Canonical correspondence analysis (CCA) was used to evaluate the possible association of gut microbes with environmental factors. In our study, the correlation between gut microbes and serum triglyceride, serum cholesterol, and LT4 doses was performed in all samples (the dose of L-thyroxine was set as zero in NC group) as well as in LRT groups, respectively. Serum triglyceride, serum cholesterol, TSH, T3, and T4 were targeted to analyze the potential influences to the microbes in the NC group. In the genus and family level, the angle between the vector of serum cholesterol and L-thyroxine (LT4) dosage was shown as a sharp angle, which implied the synergistic effect of these two factors on shaping the gut microbial profile in all samples. Serum triglycerides did not show relative correlations with either cholesterol or LT4 to the microbial community. Similar phenomena were also found in the LRT group (Figure 6). In the NC group, serum cholesterol and self-secreted T4 showed opposite effects in the regulation of the gut microbial community (obtuse angle between these two vectors), which suggested that the “enterotype” of lower cholesterol metabolic activity usually coexisted with the “enterotype” of lower thyroxine metabolic activity. Together, these results implied that cholesterol metabolism might have certain correlation with thyroid metabolism, but the detailed mechanisms need further discussion.

## Discussion

Several concomitant factors are associated with thyroid dysfunction, such as metabolic disorders including dyslipidemia and obesity. It had been widely reported that thyroid dysfunction is always associated with disorders of serum lipid profiles. The main reason might be due to abnormal regulation of thyroxine or thyrotropin to lipid metabolism of targeted tissues, such as liver or adipose (Rodondi et al., 2010; McQuade et al., 2011; Gao et al., 2013; Minarikova et al., 2014). On the other hand, our previous reports had established the potential effects of excess lipid deposition to thyroid function, by which the probable mechanism was lipotoxicity-related endoplasmic reticulum (ER) stress or mitochondrial stress of thyrocytes, subsequently leading to thyroid dysfunction. This is an innovative point of view that had been confirmed in clinical study and laboratory research (Zhao et al., 2016, 2018; Zhang et al., 2019). In this study, gut microbes, which had already been recognized as another important “endocrine factors,” was the focus to discover the possible association with SCH on clinical levels. Our findings had illustrated that the discrepancies in L-thyroxine doses required to maintain the stability of TSH level in SCH patients may be related to the different compositions of gut microbiota among subjects. The potential mechanism may be related to the variations in thyroxine metabolic capacity in the gut. Moreover, the metabolic similarity of iodothyronines and bile acid in the intestine also provides a possibility for the correlation between the host's thyroxine and cholesterol levels.

Our results revealed that the microbial diversity analysis, shown by Shannon and Simpson index, and the structure of microbes were not obviously changed according to single-factor disorder, such as serum TG, TC, or L-thyroxine treatment. These results were in slightly inconsistence with common views. It was generally accepted that microbiota in subjects with obesity or metabolic syndrome showed a typical microbial “enterotypes,” with a decreased microbial diversity (Turnbaugh et al., 2009; Sanna et al., 2019). The possible explanation might be the pathological background of SCH. SCH is an endogenous cause of serum lipid abnormalities but not the external factors such as dietary habit, which is widely accepted as an important factor in regulating lipid profiles and, more seriously, in gut microbiota. Our research is to establish the association of gut microbiota and serum lipid profile. For this purpose, subjects with medication history other than improving dyslipidemia were not excluded, which is one of the limitations of our study, and led to no significant difference in BMI levels among groups with different lipid profiles. The average FPG and blood pressure in our population are slightly higher but still within normal ranges. The reason might be that the average age of our cohort is 64.0 ± 8.3, which is relatively high in correspondence with metabolic disorders, such as serum lipid and serum glucose abnormalities. It is reported that the average blood pressure was higher in population of China's rural area (Du et al., 2019). In our study, it might be due to the higher average ages of our cohort or the normal diet habits in the countryside of northern China, which consisted of high salt, high oil recipe. Our study also performed the analysis for the population after removing T2D and/or hypertension medication cases and found out that the excluded cases were not that significant. The reason might be due to the similar prevalence of those cases among different groups. It turned out that an abnormal serum lipid profile might not influence the gut microbial community. Positive discrepancies in gut microbes might appear in the condition setting diet or BMI as the single variations but not serum lipid profiles.

Our discrepancy analysis among the LRT and NC groups and within the LRT group had helped us to explore the causal relationship between the drug and gut microbes. From beta diversity analysis between the LRT and NC groups, it can be concluded that the drug showed no obvious effect in shaping gut microbiota. However, this conclusion is not supported by the results of the analysis among H, M, and L groups, which had implied that L-thyroxine might regulate microbial profile in a dose-dependent way. In other words, subjects with a higher demand of the drug may share similar gut microbial profile. From this point, it can be partially speculated that, in the correlation between gut microbiota and thyroxine metabolism, bacteria may act as the causal factor.

In order to find more information for discussing the possible causal relationship between gut microbiota and SCH, we subsequently performed microbial analysis between L-D and L-ND groups, with the purpose to build the relevance of gut microbial profile and the SCH development. However, the results displayed by PCA or PCoA were not that obvious. The differences were not as obvious as that in other metabolic factors such as diet, for the reason that the key members of microbes participating in thyroxine metabolism might not be predominant components in shaping microbial profiles. The detail discrepancies should be analyzed in the taxa level. Relative abundance of the species with the hydrolytic activities or carbohydrate metabolic activities were changed among groups. These discrepancies may influence the metabolism of glucuronidated and sulfated iodothyronines or the regulation of intestinal barrier function. The relative abundance of the genera *Odoribacter* and *Enterococcus* were increased according to the dosage increase in L-thyroxine. *Enterococcus* is a Gram-positive, facultative anaerobic genus of the phylum *Firmicutes* with high level of intrinsic antibiotic resistance (Fisher and Phillips, 2009; Palermo et al., 2011). It was reported that *Enterococcus* increased in hypothyroid, which might be a risk factor for acquiring infections (Zhou et al., 2014). The relative abundance of the genus *Odoribacter* had been reported with a negative correlation with lipid profiles especially cholesterol level, as well as a positive correlation with other related metabolic parameters such as the body fat percentage, adiposity index, and visceral fat (Granado-Serrano et al., 2019; Sun et al., 2019). The relative abundance of the genera *Alisipes, Ruminococcus*, and *Anaerotruncus* showed discrepancies with statistical significance between L-D and L-ND groups. β-Glucuronidase activity has been characterized for the first time from *Ruminococcus gnavus* E1, an anaerobic bacterium belonging to the genus *Ruminococcus*, a dominant human gut microbiota (Beaud et al., 2005). It was reported that the abundance level of *Ruminococcus* was increased in patients with Hasmoto's thyroiditis. The genus *Alistipes* was reported to have a direct association with β-glucuronidase or β-glucosidase activity from human fecal samples (Flores et al., 2012; Chan et al., 2016). From the GeneBank database, the complete genome of one species, *Alistipes shahii* WAL 8301 (Accession number FP929032), which was isolated from human gut, contains several copies of β-glucuronidase gene. *Anaerotruncus* is isolated from human fecal samples (Lawson et al., 2004); one of its members, *Anaerotruncus massiliensis*, is isolated from an obese patient after bariatric surgery (Togo et al., 2016). It was widely accepted that *Anaerotruncus* species might be optimal probiotic strains. These species express enzymes that favor the production of butyrate (Polansky and Javaherian, 2015). Butyrates are short-chain fatty acid derivatives that are reported as important nutrients that participate in colon inflammation, with effects of stabilizing intestinal permeability (Donohoe et al., 2011).

Moreover, it was reported that the genus *Alistipes* was involved in bile acid metabolism (Huang et al., 2019). Primary bile acid was converted from cholesterol in the liver and then conjugated to taurine or glycine and secreted into the bile by transport proteins (Thomas et al., 2008). This process was similar to iodothyronine metabolism in the liver, as iodothyronine was sulfated or glucuronidated to accelerate the deiodination of thyroxine to their inactive metabolites (Sayin et al., 2013). Several intestinal microbes are proven to facilitate the deconjugation process during the conversion from primary to secondary BAs, which in turn contribute to the reabsorption of bile acids into enterocyte through the enterohepatic circulation (EHC) in the distal ileum (Zollner et al., 2006).

Considering the catalytical similarity in the hydrolytic activities of gut microbes, it may be possible to establish a relationship between thyroxine and bile acids metabolism in the gut. CCAs were performed to analyze the correlation between the flora and environmental factors (the clinical indicators of subjects collected from our survey) and found that, in the LRT group, serum cholesterol levels and L-thyroxine doses show synergistic changes with gut microbiota. In the NC group, serum cholesterol levels and T4 levels show opposite relationship with gut microbiota. This interesting phenomenon illustrated that the high level of serum cholesterol, which is the raw material for bile acids synthesis, and partially acted as the “readout” of dietary habit, could influence the community structure of gut microbiota. Conjugated bile acid and conjugated thyroxine might work together to shape the gut microbial community as the same “enterotypes,” or else disordered cholesterol metabolism might reduce the hydrolysis and reabsorption of thyroxine. This phenomenon might be displayed as a higher L-thyroxine requirement with purpose to maintain TSH levels when occurs in subjects of LRT group, whereas it would be the decrease level of T4 in the NC group.

In this study, we intend to build a relationship between SCH and dyslipidemia from the perspective of gut microbes on clinical levels. We did not find the obvious correlation between the difference in serum lipid and gut microbes; one limitation is that the lipid data of our population might be influenced by some other drug intervention such as metformin or glipizide. Interestingly, L-thyroxine doses, which are required to maintain TSH level stability in SCH patients, may be related to the different composition of gut microbiota among subjects. The underlying mechanism may be due to the differences in thyroxine metabolic capacity in the gut. In addition, the metabolic similarity of iodothyronines and bile acid in the intestine also provides possibilities for the correlation between the host's thyroxine and cholesterol levels. One limitation is that we did not find more thyroid metabolic-related species other than those lower abundance ones; it should be deeply analyzed through genomic sequencing, either in clinical sample or animal model, which would be less deviation among individuals. Another limitation of our data is that the serum lipid profiles might be influenced by other factors that we did not exclude; this might be the reason that the microbial difference among groups divided by serum lipid was not obvious.

## Data Availability Statement

The raw data supporting the conclusions of this article will be made available by the authors, without undue reservation, to any qualified researcher.

## Ethics Statement

The studies involving human participants were reviewed and approved by Ethics Committee of Shandong Provincial Hospital. The patients/participants provided their written informed consent to participate in this study.

## Author Contributions

TB and LG designed the research and wrote the paper. TB and ZY performed the study. MZ designed the clinical strategy. YG and QW performed the sample collection. WC performed the data analysis. All authors contributed to the article and approved the submitted version.

## Conflict of Interest

The authors declare that the research was conducted in the absence of any commercial or financial relationships that could be construed as a potential conflict of interest.

## Supplementary Material

Supplementary Figure 1 Curve and Bar plot analysis of Alpha diversity analysis within different groups. **(A)** Observed species curve is generated by setting the number of observed species as y-axis. The value in bar plot chart is calculated by setting average number of each group's observed species as y-axis. **(B)** Chao1 curve is generated by setting Chao1 index as y-axis. The curve reflected the relationship between the quantity of observed species and sequences. The value in bar plot chart is generated by setting average Chao1 index of each group as y-axis. The “plateaued” shape of the curve indicated that enough sequences were obtained to cover the majority of species. Shannon **(C)** and Simpson **(D)** curve is generated by setting Shannon and Simpson index as *y*-axis, respectively. Bar plots are generated by setting average Shannon and Simpson indexes of each group as *y*-axis, respectively. Higher Shannon and Simpson index indicate higher diversity. The number of sequence is set as x-axis. Each color represents one sample.

Supplementary Figure 2 Bar plot analysis of Alpha diversity analysis within different groups divided by L-thyroxine treatment or not. Observed species value **(A)** is calculated by setting average number of each group's observed species as y-axis. Chao1 value **(B)** is generated by setting average Chao1 index of each group as y-axis. Shannon **(C)** and Simpson **(D)** are generated by setting average Shannon and Simpson indexes of each group as *y*-axis, respectively. Higher Shannon and Simpson index indicate higher diversity. Each color represents one group.

Supplementary Figure 3 Principle Component Analysis among different groups divided by lipid profiles in different levels other than genus (family, order, class and phylum) level. X axis and Y axis represent the first principal component (PCA1) and the second principal component (PCA2), respectively. The percentage in the brackets represents the relative contribution of the component to the total difference. Taxa name was listed in the upper part of each diagram. Each sample was corresponded to one dot in the graph. Different group is represented by different color.

Supplementary Figure 4 Bar plot analysis of Alpha diversity analysis within groups divided by L-thyroxine dosage within LRT group. Observed species value **(A)** is calculated by setting average number of each group's observed species as y-axis. Chao1 value **(B)** is generated by setting average Chao1 index of each group as y-axis. Shannon **(C)** and Simpson **(D)** are generated by setting average Shannon and Simpson indexes of each group as *y*-axis, respectively. Higher Shannon and Simpson index indicate higher diversity. Each color represents one group.

Supplementary Figure 5 Principle Component Analysis among different groups divided by L-thyroxine treatment or not, in different levels other than genus (family, order, class and phylum) level. X axis and Y axis represent the first principal component (PCA1) and the second principal component (PCA2), respectively. The percentage in the brackets represents the relative contribution of the component to the total difference. Taxa name was listed in the upper part of each diagram. Each sample was corresponded to one dot in the graph. Different group is represented by different color.

Supplementary Figure 6 Principle Component Analysis among different groups divided by L-thyroxine dosage within LRT group, in different levels other than genus (family, order, class and phylum) level. X axis and Y axis represent the first principal component (PCA1) and the second principal component (PCA2), respectively. The percentage in the brackets represents the relative contribution of the component to the total difference. Taxa name was listed in the upper part of each diagram. Each sample was corresponded to one dot in the graph. Different group is represented by different color.

Supplementary Figure 7 Principle Component Analysis among different groups divided by **(A)** lipid profile, **(B)** L-thyroxine treatment or not, **(C)** L-thyroxine dosage within LRT group, **(D)** the development of L-thyroxine dosage within LRT group, in genus levels of the population after excluding T2D and/or hypertension medication cases. X axis and Y axis represent the first principal component (PCA1) and the second principal component (PCA2), respectively. The percentage in the brackets represents the relative contribution of the component to the total difference. Each sample was corresponded to one dot in the graph. Different group is represented by different color.

Supplementary Figure 8 Principle Co-ordination Analysis among different groups divided by **(A)** lipid profile, **(B)** L-thyroxine treatment or not, **(C)** L-thyroxine dosage within LRT group, **(D)** the development of L-thyroxine dosage within LRT group, in genus level of the population after excluding T2D and/or hypertension medication cases. X axis and Y axis represent the first principal component (PCoA1) and the second principal component (PCoA2), respectively. The percentage in the brackets represents the relative contribution of the component to the total difference. Each sample was corresponded to one dot in the graph. The circle summerized the area of gathering of the dots. Different group, together with the circle, are represented by different color.

Supplementary Figure 9 Principle Component Analysis among different groups divided by the development of L-thyroxine dosage within LRT group, in different levels other than genus (family, order, class, and phylum) level. X axis and Y axis represent the first principal component (PCA1) and the second principal component (PCA2), respectively. The percentage in the brackets represents the relative contribution of the component to the total difference. Taxa name was listed in the upper part of each diagram. Each sample was corresponded to one dot in the graph. Different group is represented by different color.

Supplementary Figure 10 Relative abundance analysis of some metabolic representative species, in population after excluding T2D and/or hypertension medication cases. **(A,B)** Relative abundance of Genus *Odoribacter, Enterococcus* among groups divided by L-thyroxine dosage. **(C–E)** Relative abundance of Genus *Alistipes, Anaerotruncus, Ruminococcus* divided by L-thyroxine dosage development within LRT group. Error bars are calculated as a standard error (SEM). The differences among groups were compared using nonparametric tests. “^*^” indicated *p* < 0.05, “^**^” indicated *p* < 0.01, were defined statistically significance.
