# Supplementary material for: Relation of Gut Microbes and L-Thyroxine Through Altered Thyroxine Metabolism in Subclinical Hypothyroidism Subjects
Source: Front Cell Infect Microbiol. 2020 Sep 18;10:495. doi: 10.3389/fcimb.2020.00495 (PMC7531258; doi:10.3389/fcimb.2020.00495)
Supplement: Supplementary file 1 [file Table_1.docx]

Supplementary Table 1. Basal physiological data analysis in population excluding T2D and/or hypertension medication cases.

| Group | Age  (year) | Gender | SBP | DBP | BMI  (kg/m^2^) | FPG  (mmol/L) | ALT  (U/L) | AST  (U/L) | TG  (mmol/L) | CHOL  (mmol/L) |
| --- | --- | --- | --- | --- | --- | --- | --- | --- | --- | --- |
| LRT | 64.8±7.3 | 8/21 | 123.4±14.3 | 75.3±10.7 | 25.2±3.4 | 5.80 (0.76) | 20.0 (10.5) | 26.0 (9.5) | 1.51 (1.19) | 5.63±1.13 |
| NC | 63.9±9.6 | 11/23 | 130.0±16.0 | 78.3±12.3 | 24.9±3.0 | 5.70 (1.03) | 18.0 (11.5) | 26.0 (9.5) | 1.29 (0.81) | 5.10±0.96 |
|  |  |  |  |  |  |  |  |  |  |  |
| S | 63.6±9.5 | 13/15 | 128.7±15.8 | 77.8±12.6 | 25.5±3.5 | 5.64 (0.48) | 18.0 (11.5) | 25.0 (7.5) | 1.06 (0.71) | 4.82±0.73 |
| STC | 65.5±7.7 | 2/15 | 124.2±14.2 | 74.3±10.1 | 24.8±2.7 | 5.76 (0.63) | 18.0 (9.0) | 27.0 (8.0) | 1.28 (0.44) | 6.22±0.67* |
| STG | 64.0±7.9 | 4/7 | 132.0±17.3 | 83.2±11.2 | 24.9±3.5 | 6.20 (1.34) | 21.0 (18.0) | 28.0 (15.0) | 2.21 (0.76)* | 4.51±0.84 |
| SM | 64.2±9.5 | 0/7 | 118.8±13.1 | 69.5±7.5 | 24.1±3.0 | 5.90 (1.01) | 23.0 (15.0) | 29.0 (14.0) | 2.86 (1.00)* | 6.60±0.65* |
|  |  |  |  |  |  |  |  |  |  |  |
| H | 64.0±4.9 | 3/5 | 129.3±12.0 | 78.0±4.2 | 25.1±2.6 | 5.63 (1.50) | 20.5 (10.0) | 27.5 (12.8) | 1.51 (1.28) | 5.79±1.48 |
| M | 65.4±8.8 | 3/12 | 123.1±17.8 | 75.0±12.9 | 25.5±3.7 | 6.00 (0.59) | 18.0 (18.0) | 24.0 (9.0) | 1.51 (1.65) | 5.45±0.95 |
| L | 65.3±6.8 | 2/5 | 122.6±13.3 | 73.9±10.7 | 24.0±3.6 | 5.80 (0.30) | 20.0 (8.0) | 26.0 (9.0) | 1.40 (0.74) | 5.94±1.08 |
|  |  |  |  |  |  |  |  |  |  |  |
| L-D | 65.3±7.0 | 5/18 | 123.6±12.8 | 73.3±8.8* | 24.8±3.0 | 5.80 (0.75) | 18.0 (11.0) | 26.0 (11.0) | 1.50 (0.86) | 5.65±1.10 |
| L-ND | 64.2±8.9 | 3/4 | 127.7±23.2 | 83.5±13.5 | 26.0±4.7 | 5.80 (0.96) | 21.0 (14.0) | 28.0 (8.0) | 1.70 (1.90) | 5.66±1.25 |

Gender, data was shown as male/female.

BMI, body mass index.

DM, Type 2 diabetes Millitus.

FPG, fasting plasma glucose.

TG, serum triglyceride

CHOL, serum cholesterol

AST, [glutamic-pyruvic transaminase](http://www.baidu.com/link?url=ZP37C3S25A2fLbPvxMI3fbsVKo0Mz0vMMsl_GrGmiVy3E52NYDaDn2cRn-dLnnbs94kwTQ_ZiFsm7skHIRwiE8m9EVkyH4sTCfySK0Dg-5iGjq4pP-0tKILYboro2CYClkF9CPigNqTbSLdjrM7QBK)

ALT, [glutamic oxaloacetic transaminase](http://www.baidu.com/link?url=n-R9EFTmnueVCFDvqpA5PJ39FUeaQH04sk_YOQ7pFQGAV_xyQYPgVP_7KtYGW0O5zU2o4KGSqCx2_X752GTNTF3w3-mvGt68RulcFT2cCU0eOV9o2b0DYJpyF8YdFeBMRdV52xb1z7_jbidS5oPyr_)

*, p<0.01 within their respective groups.
