# Supplementary figures and images for: Relation of Gut Microbes and L-Thyroxine Through Altered Thyroxine Metabolism in Subclinical Hypothyroidism Subjects
Source: Front Cell Infect Microbiol. 2020 Sep 18;10:495. doi: 10.3389/fcimb.2020.00495 (PMC7531258; doi:10.3389/fcimb.2020.00495)

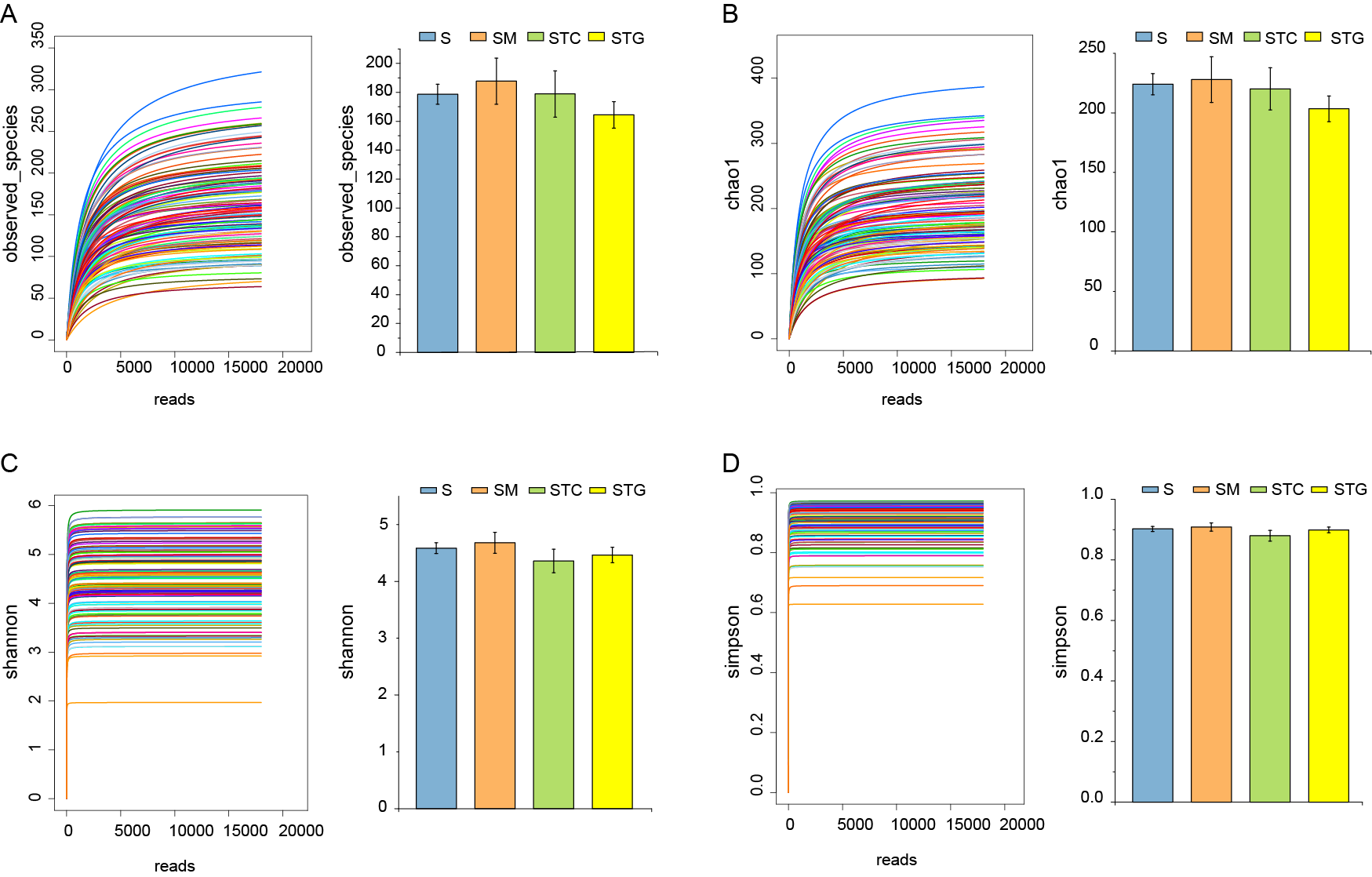

Supplement: Supplementary Figure 1 — Curve and Bar plot analysis of Alpha diversity analysis within different groups. (A) Observed species curve is generated by setting the number of observed species as y-axis. The value in bar plot chart is calculated by setting average number of each group's observed species as y-axis. (B) Chao1 curve is generated by setting Chao1 index as y-axis. The curve reflected the relationship between the quantity of observed species and sequences. The value in bar plot chart is generated by setting average Chao1 index of each group as y-axis. The “plateaued” shape of the curve indicated that enough sequences were obtained to cover the majority of species. Shannon (C) and Simpson (D) curve is generated by setting Shannon and Simpson index as y-axis, respectively. Bar plots are generated by setting average Shannon and Simpson indexes of each group as y-axis, respectively. Higher Shannon and Simpson index indicate higher diversity. The number of sequence is set as x-axis. Each color represents one sample. [file Image_1.TIF]

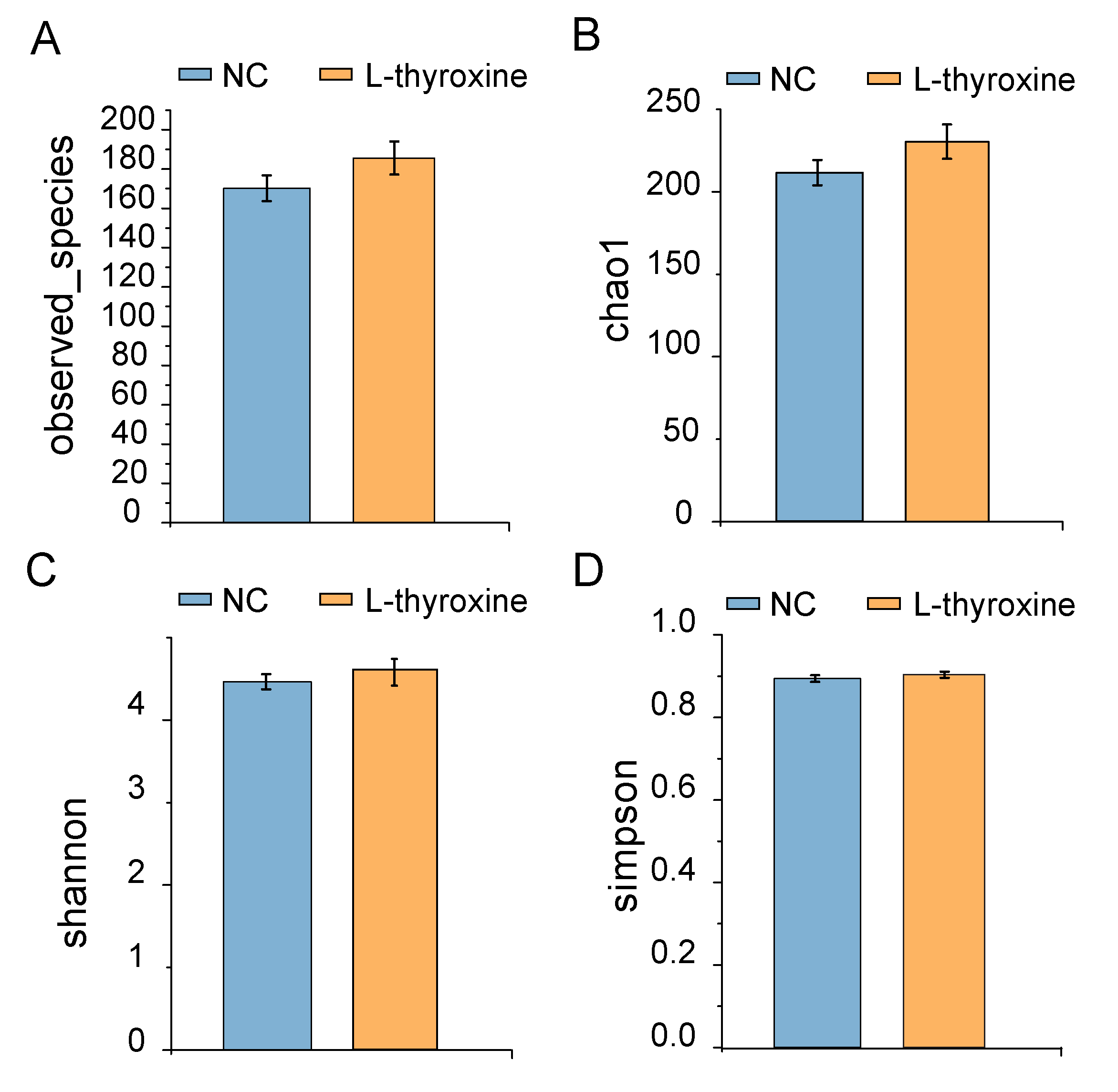

Supplement: Supplementary Figure 2 — Bar plot analysis of Alpha diversity analysis within different groups divided by L-thyroxine treatment or not. Observed species value (A) is calculated by setting average number of each group's observed species as y-axis. Chao1 value (B) is generated by setting average Chao1 index of each group as y-axis. Shannon (C) and Simpson (D) are generated by setting average Shannon and Simpson indexes of each group as y-axis, respectively. Higher Shannon and Simpson index indicate higher diversity. Each color represents one group. [file Image_2.TIF]

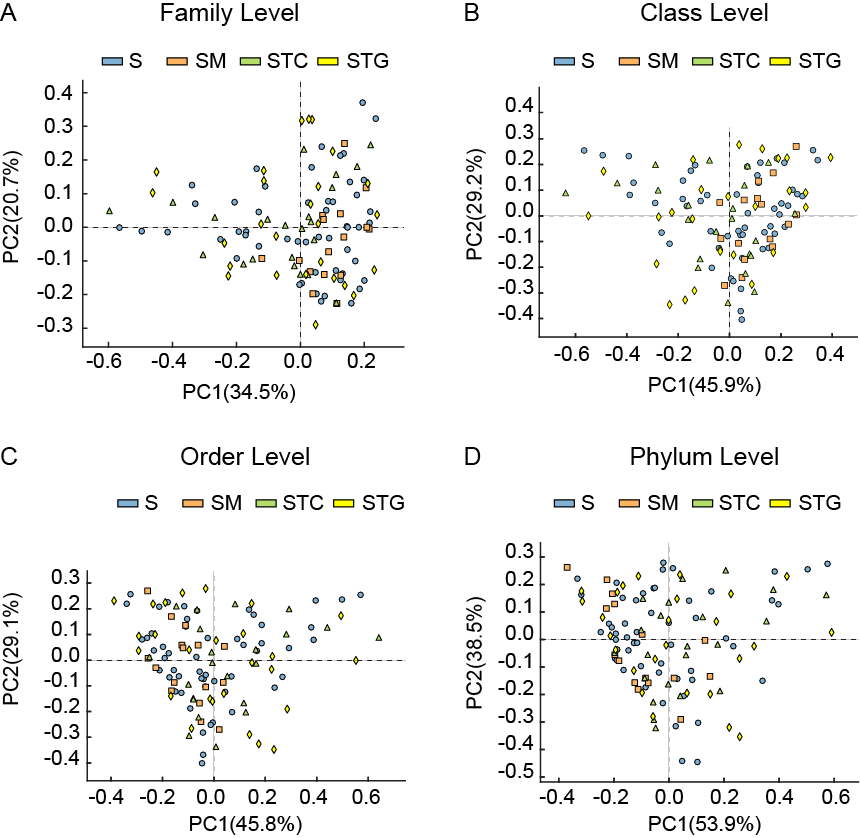

Supplement: Supplementary Figure 3 — Principle Component Analysis among different groups divided by lipid profiles in different levels other than genus (family, order, class and phylum) level. X axis and Y axis represent the first principal component (PCA1) and the second principal component (PCA2), respectively. The percentage in the brackets represents the relative contribution of the component to the total difference. Taxa name was listed in the upper part of each diagram. Each sample was corresponded to one dot in the graph. Different group is represented by different color. [file Image_3.TIF]

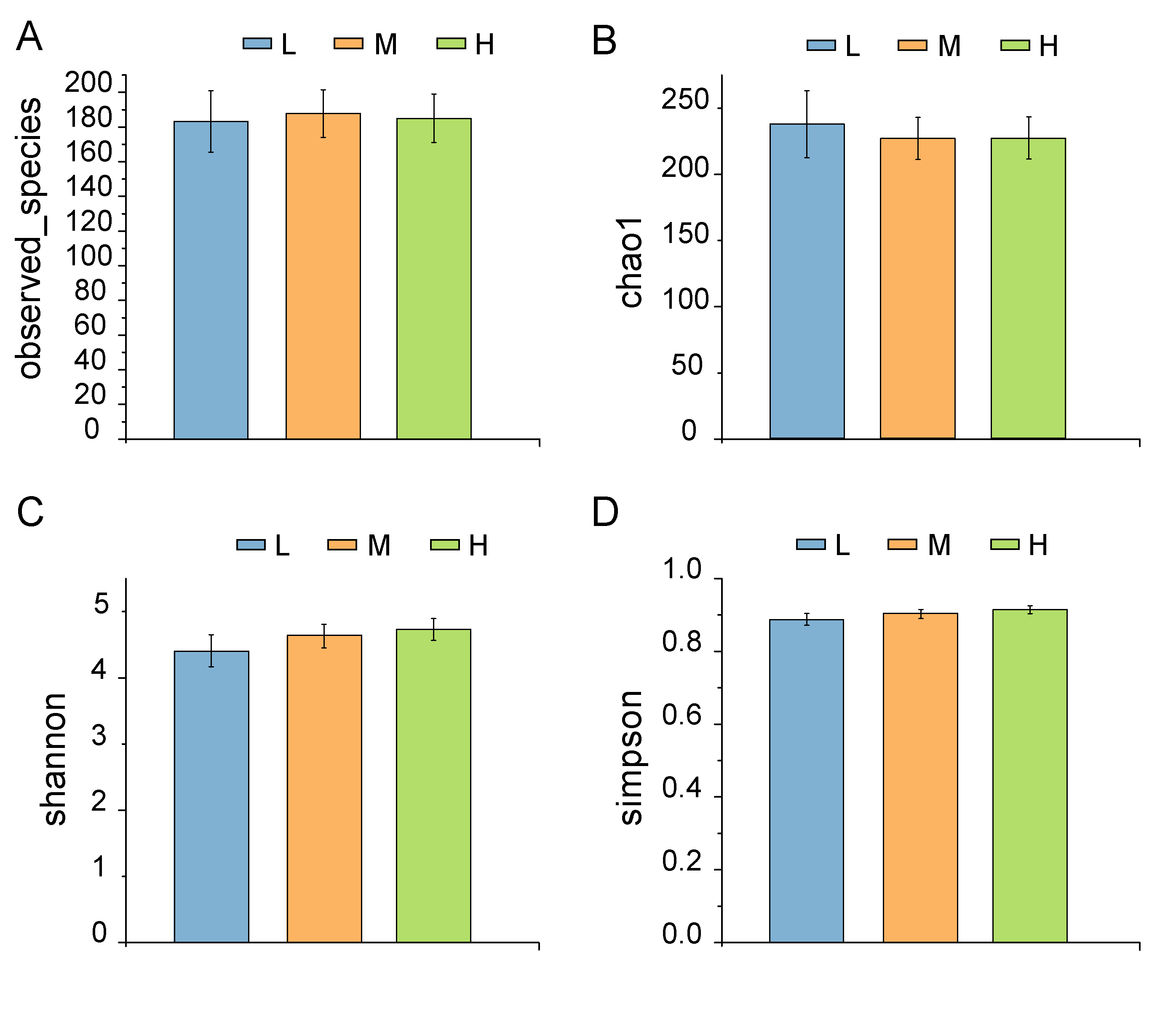

Supplement: Supplementary Figure 4 — Bar plot analysis of Alpha diversity analysis within groups divided by L-thyroxine dosage within LRT group. Observed species value (A) is calculated by setting average number of each group's observed species as y-axis. Chao1 value (B) is generated by setting average Chao1 index of each group as y-axis. Shannon (C) and Simpson (D) are generated by setting average Shannon and Simpson indexes of each group as y-axis, respectively. Higher Shannon and Simpson index indicate higher diversity. Each color represents one group. [file Image_4.TIF]

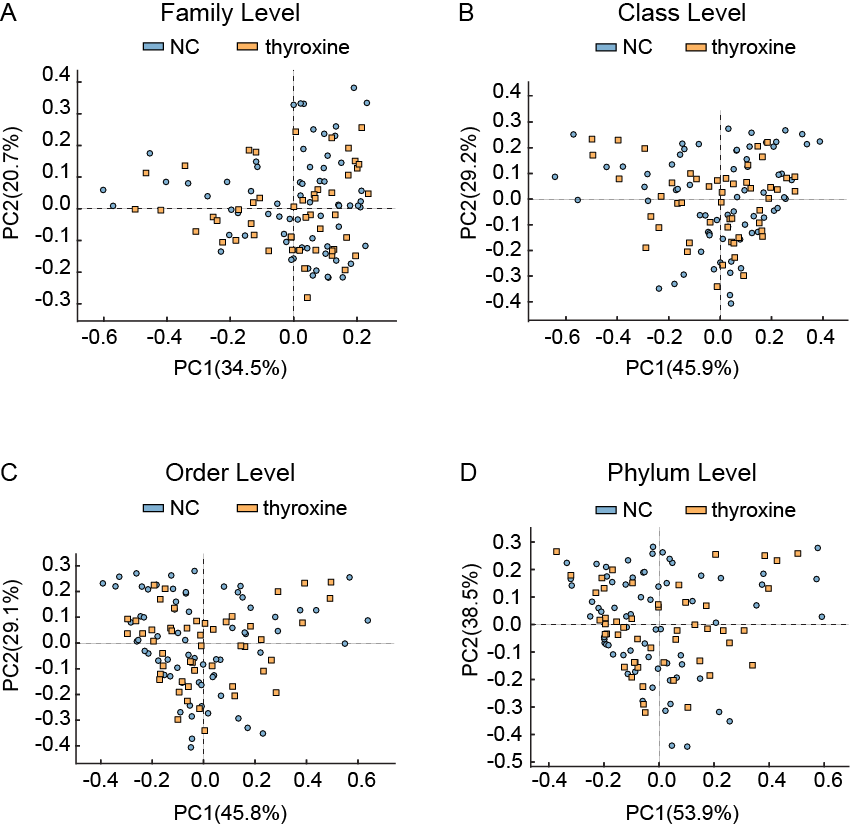

Supplement: Supplementary Figure 5 — Principle Component Analysis among different groups divided by L-thyroxine treatment or not, in different levels other than genus (family, order, class and phylum) level. X axis and Y axis represent the first principal component (PCA1) and the second principal component (PCA2), respectively. The percentage in the brackets represents the relative contribution of the component to the total difference. Taxa name was listed in the upper part of each diagram. Each sample was corresponded to one dot in the graph. Different group is represented by different color. [file Image_5.TIF]

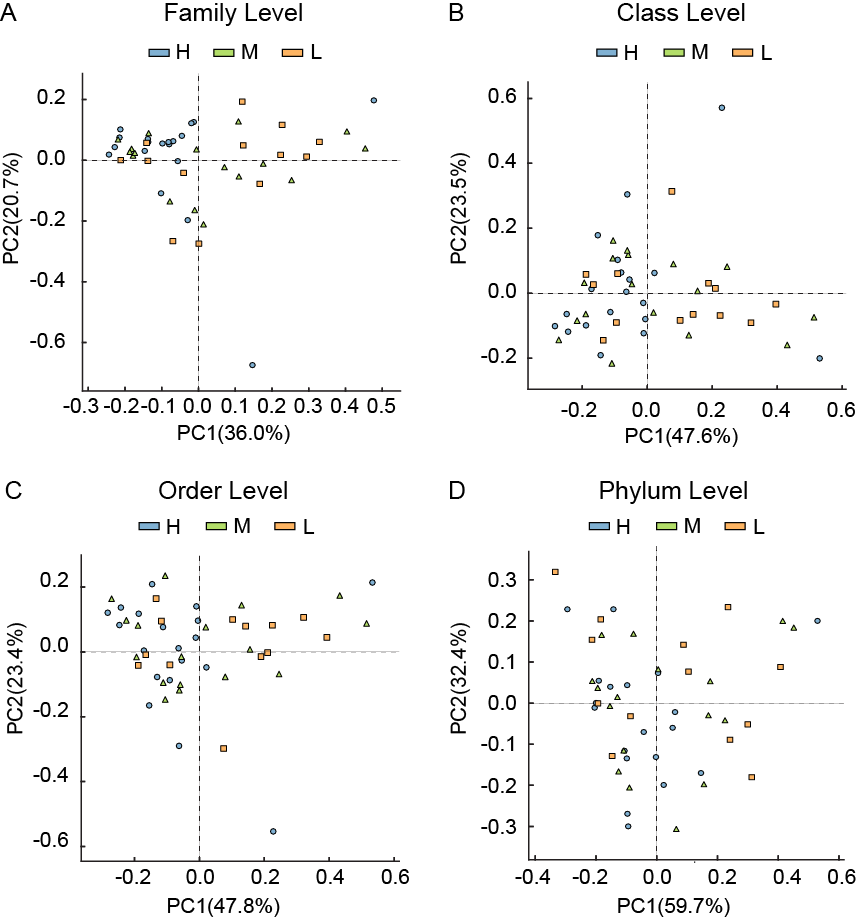

Supplement: Supplementary Figure 6 — Principle Component Analysis among different groups divided by L-thyroxine dosage within LRT group, in different levels other than genus (family, order, class and phylum) level. X axis and Y axis represent the first principal component (PCA1) and the second principal component (PCA2), respectively. The percentage in the brackets represents the relative contribution of the component to the total difference. Taxa name was listed in the upper part of each diagram. Each sample was corresponded to one dot in the graph. Different group is represented by different color. [file Image_6.TIF]

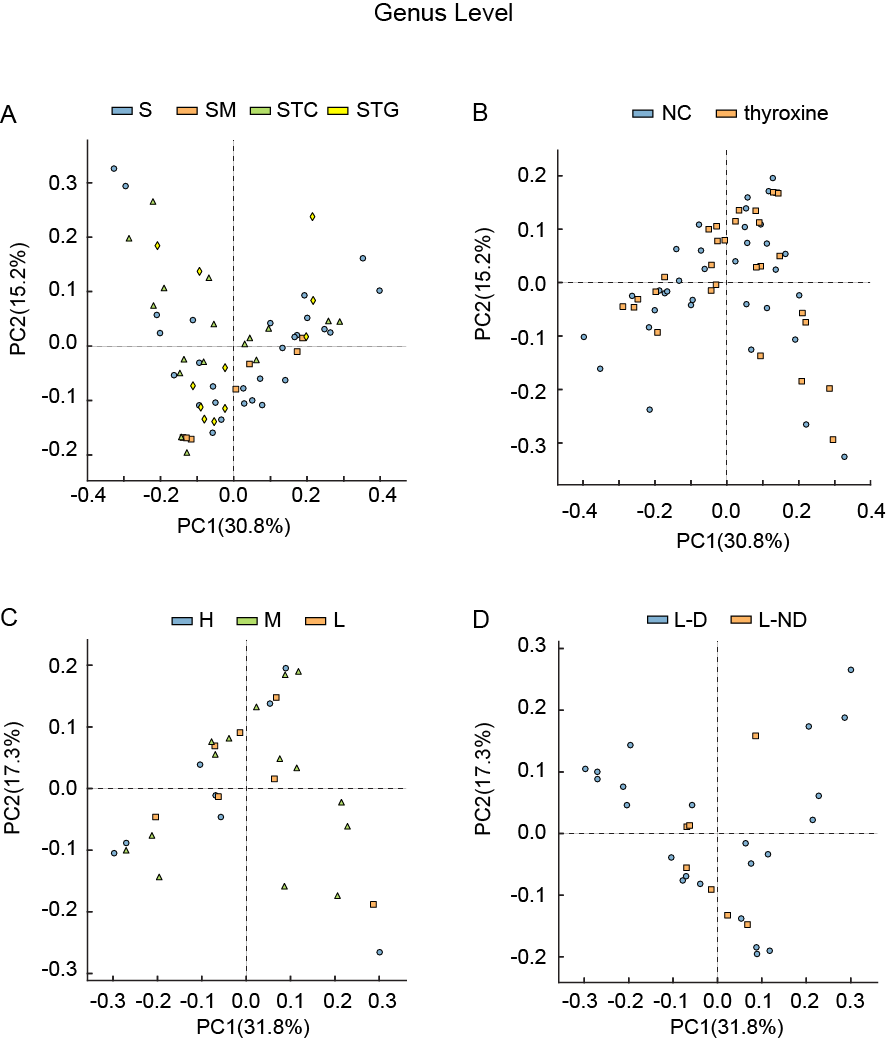

Supplement: Supplementary Figure 7 — Principle Component Analysis among different groups divided by (A) lipid profile, (B) L-thyroxine treatment or not, (C) L-thyroxine dosage within LRT group, (D) the development of L-thyroxine dosage within LRT group, in genus levels of the population after excluding T2D and/or hypertension medication cases. X axis and Y axis represent the first principal component (PCA1) and the second principal component (PCA2), respectively. The percentage in the brackets represents the relative contribution of the component to the total difference. Each sample was corresponded to one dot in the graph. Different group is represented by different color. [file Image_7.TIF]

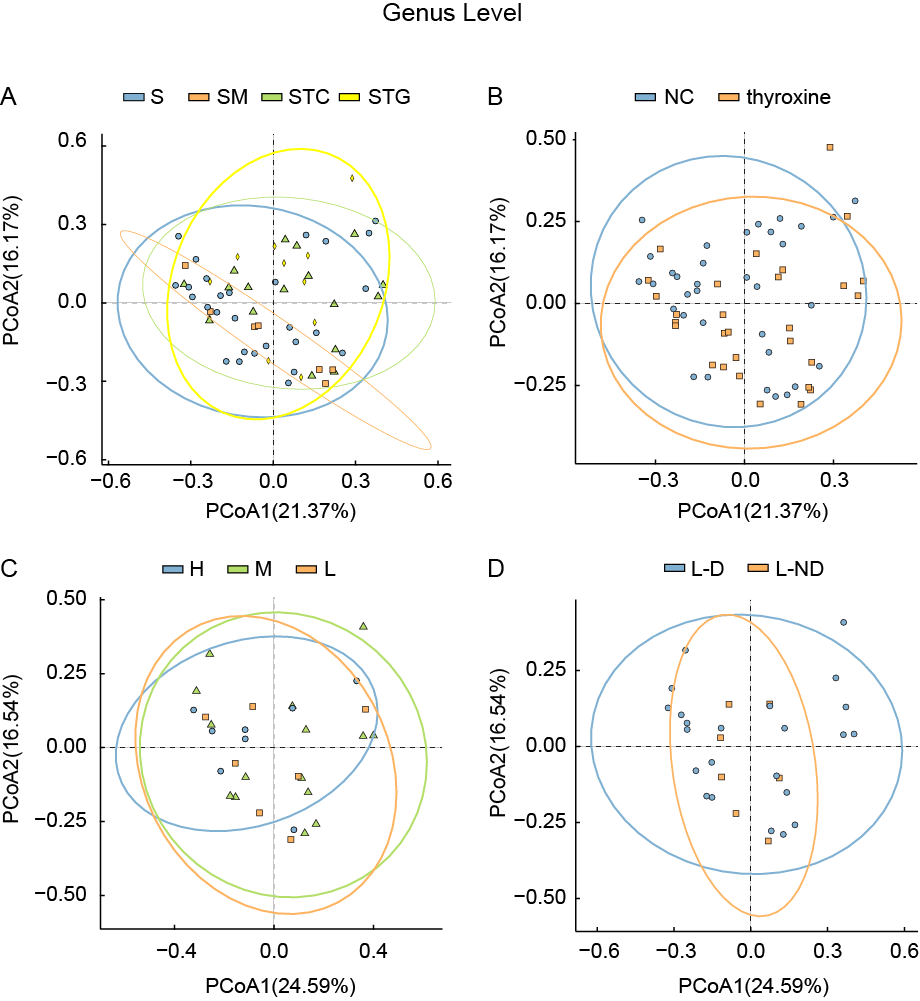

Supplement: Supplementary Figure 8 — Principle Co-ordination Analysis among different groups divided by (A) lipid profile, (B) L-thyroxine treatment or not, (C) L-thyroxine dosage within LRT group, (D) the development of L-thyroxine dosage within LRT group, in genus level of the population after excluding T2D and/or hypertension medication cases. X axis and Y axis represent the first principal component (PCoA1) and the second principal component (PCoA2), respectively. The percentage in the brackets represents the relative contribution of the component to the total difference. Each sample was corresponded to one dot in the graph. The circle summerized the area of gathering of the dots. Different group, together with the circle, are represented by different color. [file Image_8.TIF]

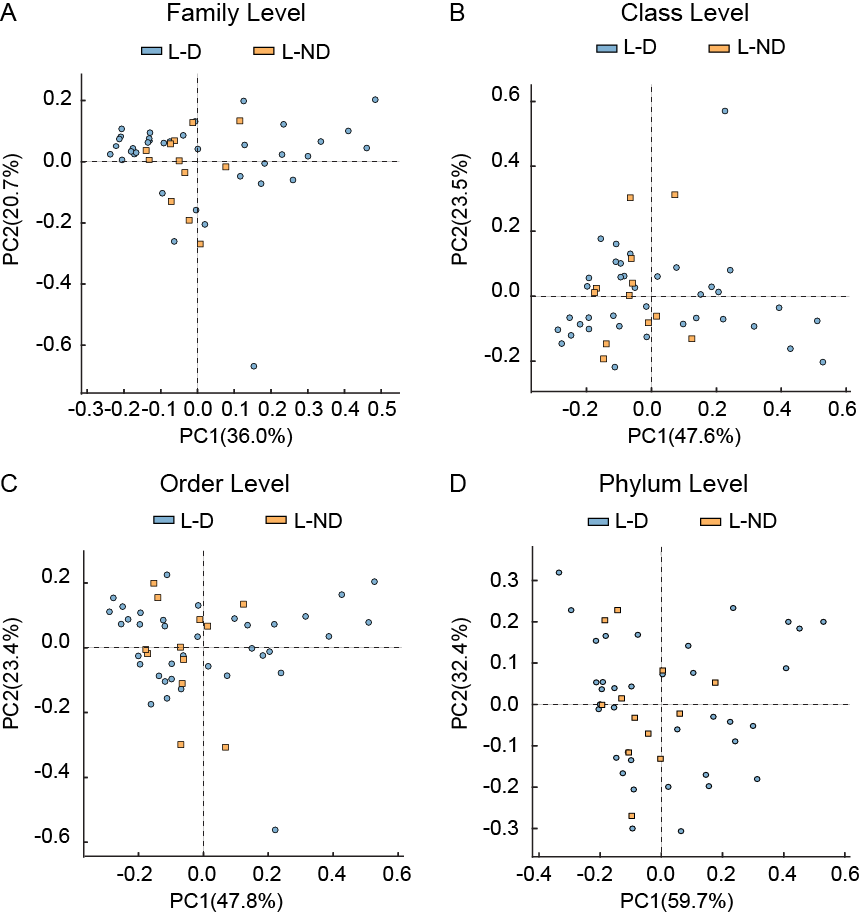

Supplement: Supplementary Figure 9 — Principle Component Analysis among different groups divided by the development of L-thyroxine dosage within LRT group, in different levels other than genus (family, order, class, and phylum) level. X axis and Y axis represent the first principal component (PCA1) and the second principal component (PCA2), respectively. The percentage in the brackets represents the relative contribution of the component to the total difference. Taxa name was listed in the upper part of each diagram. Each sample was corresponded to one dot in the graph. Different group is represented by different color. [file Image_9.TIF]

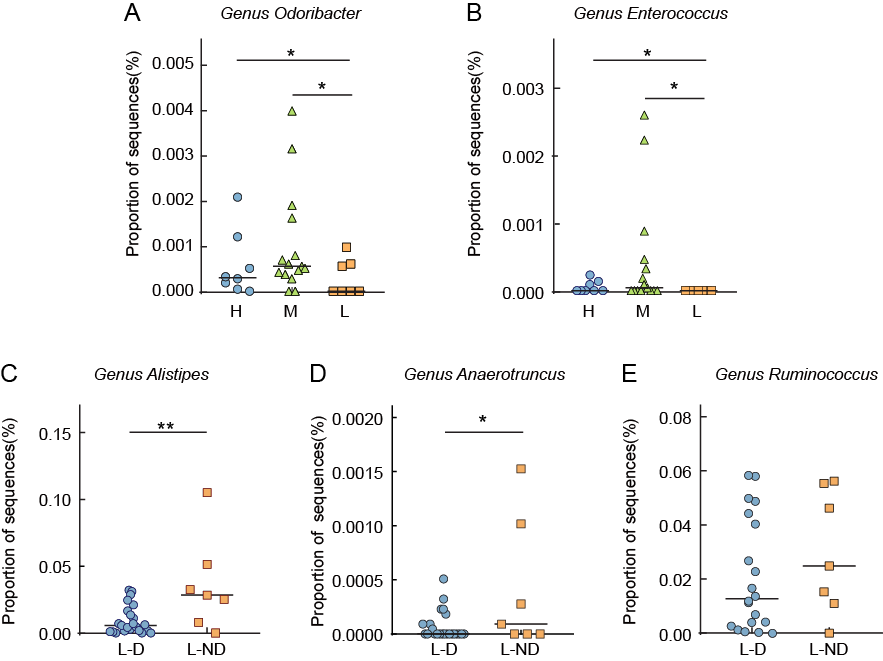

Supplement: Supplementary Figure 10 — Relative abundance analysis of some metabolic representative species, in population after excluding T2D and/or hypertension medication cases. (A,B) Relative abundance of Genus Odoribacter, Enterococcus among groups divided by L-thyroxine dosage. (C–E) Relative abundance of Genus Alistipes, Anaerotruncus, Ruminococcus divided by L-thyroxine dosage development within LRT group. Error bars are calculated as a standard error (SEM). The differences among groups were compared using nonparametric tests. “*” indicated p < 0.05, “**” indicated p < 0.01, were defined statistically significance. [file Image_10.TIF]
